# A Quinone-Based Cathode Material for High-Performance Organic Lithium and Sodium Batteries

**DOI:** 10.1021/acsaem.1c01339

**Published:** 2021-10-18

**Authors:** Dylan Wilkinson, Manik Bhosale, Marco Amores, Gollapally Naresh, Serena A. Cussen, Graeme Cooke

**Affiliations:** † School of Chemistry, University of Glasgow, Glasgow G12 8QQ, U.K.; ‡ Department of Chemical and Biological Engineering, University of Sheffield, Sheffield S1 3JD, U.K.; § Department of Materials Science and Engineering, University of Sheffield, Sheffield S1 3JD, U.K.

**Keywords:** quinone, organic cathode, Li-ion battery, Na-ion battery, stability, capacity

## Abstract

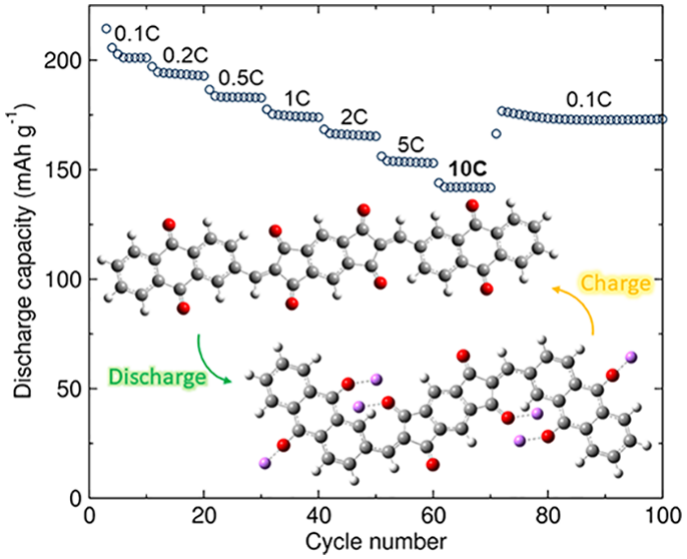

With the increased application of batteries in powering electric vehicles as well as potential contributions to utility-scale storage, there remains a need to identify and develop efficient and sustainable active materials for use in lithium (Li)- and sodium (Na)-ion batteries. Organic cathode materials provide a desirable alternative to inorganic counterparts, which often come with harmful environmental impact and supply chain uncertainties. Organic materials afford a sustainable route to active electrodes that also enable fine-tuning of electrochemical potentials through structural design. Here, we report a bis-anthraquinone-functionalized s-indacene-1,3,5,7(2H,6H)-tetraone (**BAQIT**) synthesized using a facile and inexpensive route as a high-capacity cathode material for use in Li- and Na-ion batteries. **BAQIT** provides multiple binding sites for Li- and Na-ions, while maintaining low solubility in commercial organic electrolytes. Electrochemical Li-ion cells demonstrate excellent stability with discharge capacities above 190 mAh g^–1^ after 300 cycles at a 0.1C rate. The material also displayed excellent high-rate performance with a reversible capacity of 142 mAh g^–1^ achieved at a 10C rate. This material affords high power capabilities superior to current state-of-the-art organic cathode materials, with values reaching 5.09 kW kg^–1^. The Na-ion performance was also evaluated, exhibiting reversible capacities of 130 mAh g^–1^ after 90 cycles at a 0.1C rate. This work offers a structural design to encourage versatile, high-power, and long cycle-life electrochemical energy-storage materials.

## 1 Introduction

There is an increasing demand for energy-storage solutions that not only deliver the required energy and power densities for an application but also provide more versatile, lighter, environmentally sustainable, and economically viable approaches.^1−6^ Lithium-ion (Li-ion) batteries deliver high energy and power densities, making them the preferred technology for portable electronics and electric vehicles.^7−12^ Relying typically on inorganic intercalation-type cathodes,^13−16^ theoretical energy densities are limited by the number of redox-active sites available, and capacities are typically on the order of ∼200 mAh g^–1^. The use of toxic and/or geopolitically conflicted elements such as cobalt have given rise to alternative technologies seeking a compromise between performance and sustainability and a reduction in our reliance on elements, which present a supply chain risk. Additionally, there is a desire to improve sustainability which has seen an increased push for lower-cost sodium ion (Na-ion) alternatives or the use of sustainable and environmentally friendly organic cathode materials.^17−19^ These alternatives may also play a crucial role in grid storage, where suitable energy storage is critical for ironing out the inherent peaks and troughs associated with renewables and reducing renewable curtailment.^20,21^

Organic materials present an enticing prospect where a tunable molecular structure and high structural diversity affords advantages over inorganic counterparts.^22−31^ Organic cathode materials may comprise a range of materials, including small molecules, polymers, and covalent organic frameworks.^32,33^ Electrochemical performance can be fine-tuned through judicious choice of functional groups that contain light elements and potential group I binding sites, allowing for high energy density and flexibility. For example, the addition of carbonyl groups into the organic material opens up the possibility of inexpensive battery cathodes with theoretical capacities in excess of 300 mAh g^–1^.^4^ These attributes also make organic batteries suitable for wearable and/or portable electronics applications.^34^ The greatest hindrance and ultimately the main drawback of organic cathode materials at present are their dissolution into the liquid electrolyte. As the battery is charged and discharged through multiple cycles, organic materials tend to dissolve into the organic electrolyte, which manifests in fast capacity fading with repeated cycling.

**Figure 1:**
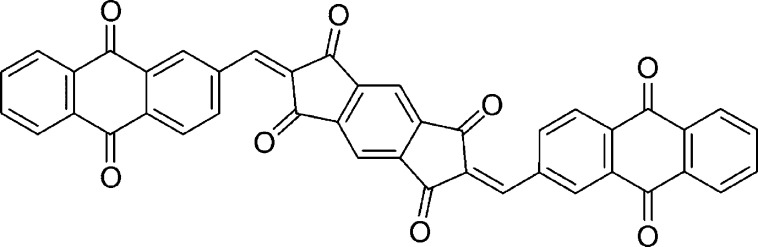
Chemical structure of **BAQIT**.

Herein, we report the synthesis of a high-capacity multicarbonyl-based cathode material featuring a s-indacene-1,3,5,7(2H,6H)-tetraone core that can be straightforwardly synthesized from pyromellitic dianhydride.^35^ The activated CH_2_ centers of the core allows for the application of Knoevenagel-like condensation reactions to be carried out to deliver fully conjugated systems, as exemplified by the previous synthesis of donor–acceptor molecules for organic photovoltaics and photophysical studies.^36−38^ In particular, we report the low-cost synthesis of the bis-anthraquinone functionalized s-indacene-1,3,5,7(2H,6H)-tetraone (**BAQIT**) cathode material (Figure 1) by functionalizing the central s-indacene-1,3,5,7(2H,6H)-tetraone moiety with two anthraquinone units to create a fully conjugated molecule featuring eight carbonyl moieties. The redox properties of anthraquinone have been widely studied, and the molecule has been shown to undergo a reversible two electron reduction at a potential ca. 2.2 V vs Li/Li^+^.^40,41^ However, the cyclability of anthraquinone as a battery cathode material is usually poor because of its dissolution into the electrolyte,^42^ which gives rise to weak capacity retention over long cycling. To overcome the solubility issues of anthraquinone, researchers have modified the molecular structure by forming polymers,^43−45^ extending the π structure^46^ or by linking anthraquinone units through bridging moieties to form dimers.^47^ The latter strategy is adopted in this study. A simple s-indacene derivative has been reported for Li-ion batteries, achieving a reversible capacity up to 50 cycles; however, this material suffers from significant capacity drop off at high C-rates.^39^

We demonstrate that by attaching two anthraquinone units to the central s-indacene-1,3,5,7(2H,6H)-tetraone core, a large elongated planar structure is formed. This affords several Li- and Na-ion binding sites while simultaneously ensuring its intractability in common liquid electrolytes, which is desirable for mitigating dissolution issues during cycling. We have investigated the sequential binding mechanism of Li^+^ and Na^+^ ions and subsequent structural transformation of **BAQIT** using density functional theory (DFT) calculations and elucidated its promising cycling performance in Li- and Na-ion half-cells.

## 2 Results and Discussion

**Figure 2:**
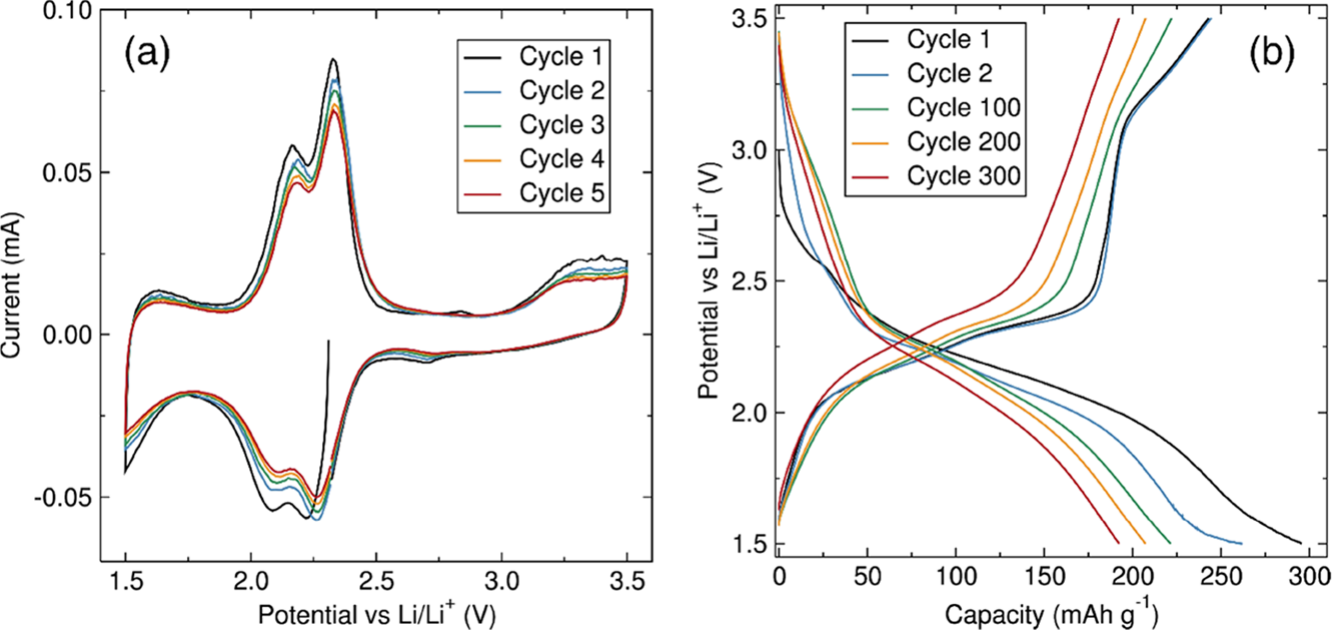
(a) CV of **BAQIT** cathode material and (b) galvanostatic cycling at room temperature at a 0.1C rate in Li half-cells.

The synthesis of **BAQIT** is described in the Supporting Information. As anticipated, the conjugated nature of this compound makes it highly insoluble in most organic solvents. In order to prove the low solubility of **BAQIT**, we have checked the solubility of **BAQIT** in dimethoxyethane (DME) and a mixture of ethylene carbonate (EC) with dimethylcarbonate (DMC) and compared it with anthraquinone (AQ) and the core indacene tetraone (IT). For this, a fixed quantity of AQ, IT, and **BAQIT** (10 mg) was separately taken in 4 mL of DME and a mixture of EC and DMC (1:1 v/v ratio), as shown in Figure S1. Also, we have checked the solubility of these electrodes in the electrolyte by ultraviolet–visible (UV–vis) spectroscopy (Figure S1). All the experimental details are described in the Supporting Information. From these experiments, we confirmed that because of the extended conjugated structure of **BAQIT**, the material displayed poor solubility in the electrolyte. We wish to exploit a property to evaluate its stability as a cathode upon repeated charge/discharge cycles in Li- and Na-ion half-cells. Cyclic voltammograms (CVs) were recorded in the potential ranges of 1.5–3.5 V vs Li/Li^+^ at a scan rate of 0.1 mV s^–1^ (Figure 2a). Two reduction peaks at 2.26 and 2.1 V and two oxidation peaks at 2.33 and 2.18 V were observed. The potential difference between the reduction peaks and the respective oxidation peaks are 70 and 80 mV, respectively, indicating a highly reversible redox process with low resistance. Six carbonyl groups are involved in the redox reaction in the studied potential range of 1.5–3.5 V, with the first of the redox peaks representing the reduction of the four carbonyl groups of the anthraquinone units, while the second redox peak represents the reduction of the two carbonyl groups of the core moiety.^43^ Reducing the lower voltage limit to 1 V to further reduce the remaining carbonyl groups resulted in the emergence of an additional irreversible peak at 1.44 V (Figure S2). This indicates that the reduction of the remaining carbonyl groups during a deep discharge process is likely accompanied by structural damage, which may be the result of additional repulsion between the injected electrons in the conjugated quinone framework, as well as possible electrolyte decomposition. Reducing the lower voltage limit further to 0.7 V shows additional irreversible peaks, as well as a large potential difference between the main oxidation and reduction redox processes. This suggests 1.5–3.5 V as an adequate cycling potential window to achieve reversible cycling performance.

**Figure 3:**
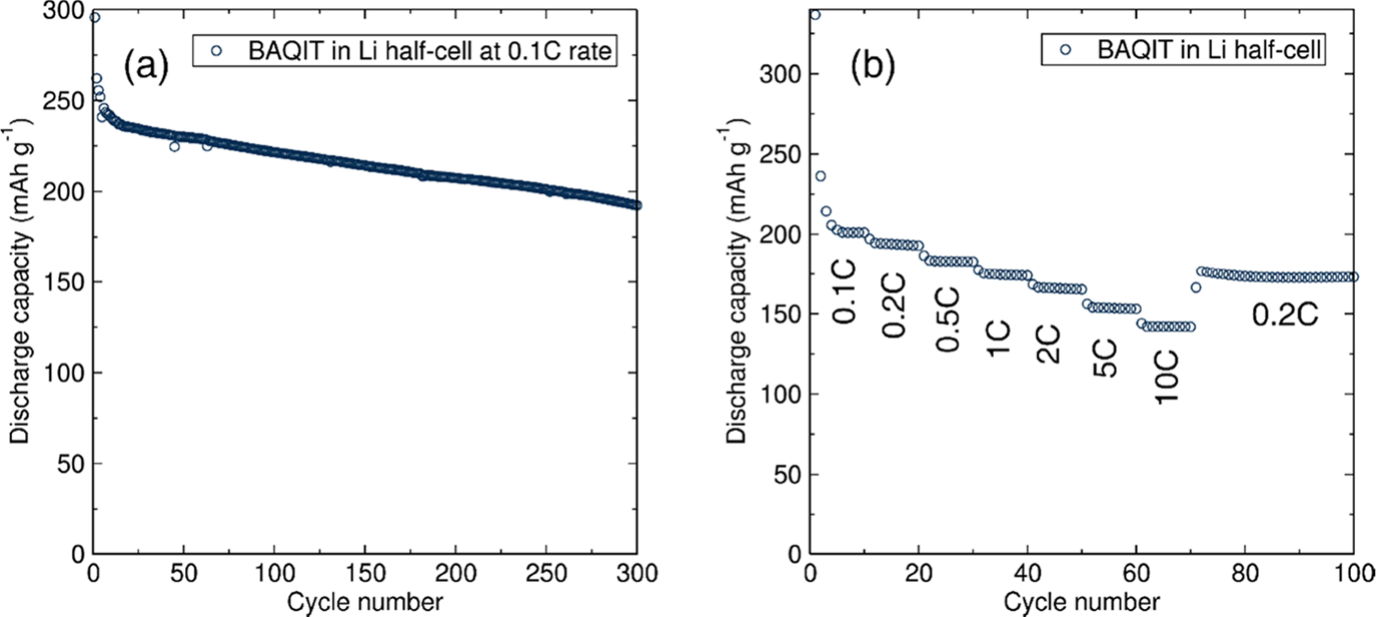
(a) Long-term galvanostatic cycling performance at 0.1C and (b) rate capability of **BAQIT** as a cathode material in Li half-cells.

Figure 2b shows the galvanostatic charge–discharge profiles of **BAQIT** at a 0.1C rate over 300 cycles. **BAQIT** delivers a first discharge capacity of 295 mAh g^–1^, with subsequent charge–discharge cycles, resulting in a reversible capacity of ∼250 mAh g^–1^, which matches the theoretical capacity of **BAQIT** for a six-electron transfer. Even after 300 cycles, the reversible capacity was still maintained above 190 mAh g^–1^, as shown in Figure 3a. The rate capability was evaluated by cycling at various specific currents from 0.1C to 10C rate (Figure 3b). **BAQIT** shows reversible capacities of 236, 195, 184, 175, 167, and 154 mAh g^–1^ at current densities of 0.1C, 0.2C, 0.5C, 1C, 2C, and 5C, respectively. More interestingly, even at a high current of 10C, corresponding to a charge or discharge step in 6 min, a reversible capacity of 142 mAh g^–1^ can be achieved. This is attributed to facile charge transport within the extended π-conjugated structure of **BAQIT**. A Ragone plot clearly evidences the high-power capabilities of the material with values near 5.09 kW kg^–1^ (Figure S3a). This value is superior to the state-of-the art nickel–manganese–cobalt (NMC) materials (≈220 mAh g^–1^)^48^ and to other high power organic cathodes such as pyrene-4,5,9,10-tetraone-containing polymers where a value of 2.9 kW kg^–1^ has been reported.^49^

Decreasing the current rate to 0.2C after fast charging/discharging exhibits a recovery of reversible capacity to 177 mAh g^–1^, suggesting excellent stability. We further analyzed the rate capability at extremely high cycling rates to assess the limits of **BAQIT** and standardize the performance relative to other cathode materials.^50^ Cycling the battery cell at low and very high cycling rates allows for the calculation of the τ and *n* parameters related to the rate at which the capacity starts to decay rapidly and how rapid that decay is, and these are illustrated in Figure S3b. These values of *τ* and *n*, 68.9 s and 0.73, respectively, were superior to those of commonly inorganic oxide materials and similar to high-rate performance electrodes for Li-ion batteries, such as NMC cathodes (*n* = 0.53–1.09, τ = 137–284 s), demonstrating the superior performance of **BAQIT** for high-power applications.^50^

EIS analyses were conducted to analyze any changes in charge-transfer or interfacial resistances of the **BAQIT** electrode upon cycling. Nyquist plots of the **BAQIT** electrode at a pristine state and after 10 charge/discharge cycles are shown in Figure S4. A semicircle in the high-frequency region originates from the charge-transfer resistance and it is followed by a tail in the low-frequency region, resulting from the resistance associated with solid-state Li-ion diffusion. The charge-transfer resistance increased after cycling from 620 to 680 Ω, again suggesting the high stability of this material and the absence of resistive interphase formation during cycling.

The long-term cycle stability of **BAQIT** was also evaluated at 0.5C and 2C cycling rates (Figure S5). After a few initial cycles at 0.5C, the capacity stabilizes to ≈180 mAh g^–1^, and a reversible capacity of 130 mAh g^–1^ is retained after 1990 cycles, demonstrating excellent cycle stability with a capacity retention of 72%. After a few initial cycles at 2C, the capacity is stabilized to ≈155 mAh g^–1^, which is retained to above 130 mAh g^–1^ after 2200 cycles with an impressive capacity retention of 86%. This demonstrates **BAQIT** as a cathode with excellent long-term cycling performance even at high rates compared to recent analogous organic cathode materials (Table S1).^51−54^ The effect of the voltage cycling window was also analyzed, and we have shown that widening the potential window to 1 V results in rapid capacity deterioration (Figure S2c).

The galvanostatic intermittent titration technique (GITT) was used to evaluate the Li-ion diffusion properties in **BAQIT** and correlate this with excellent rate capability performance (Figure S6). The calculated diffusion length (*D*/*L*^2^) across the majority of the charge and discharge profile is between 10^–5^ and 10^–4^ s^–1^. These values are larger than those reported for commercial layered oxide cathodes^55^ and is in line with other high-rate capability electrodes,^56^ underpinning one of the reasons behind the observed good rate capability of **BAQIT**.

To examine the lithiation mechanism taking place in **BAQIT**, ex situ attenuated total reflectance-infrared (ATR-IR) spectroscopy was performed at different states of charge (half discharge, full discharge, half charge, and full charge), as shown in Figure S7. A band centered at 1669 cm^–1^ with a shoulder at 1653 cm^–1^ can be ascribed to the carbonyl group stretching vibration. These two characteristic peaks gradually become weaker during the discharge process and then re-emerge gradually during charging, demonstrating that the reversible lithiation/dilithiation process is centered around the carbonyl moieties.

**Figure 4:**
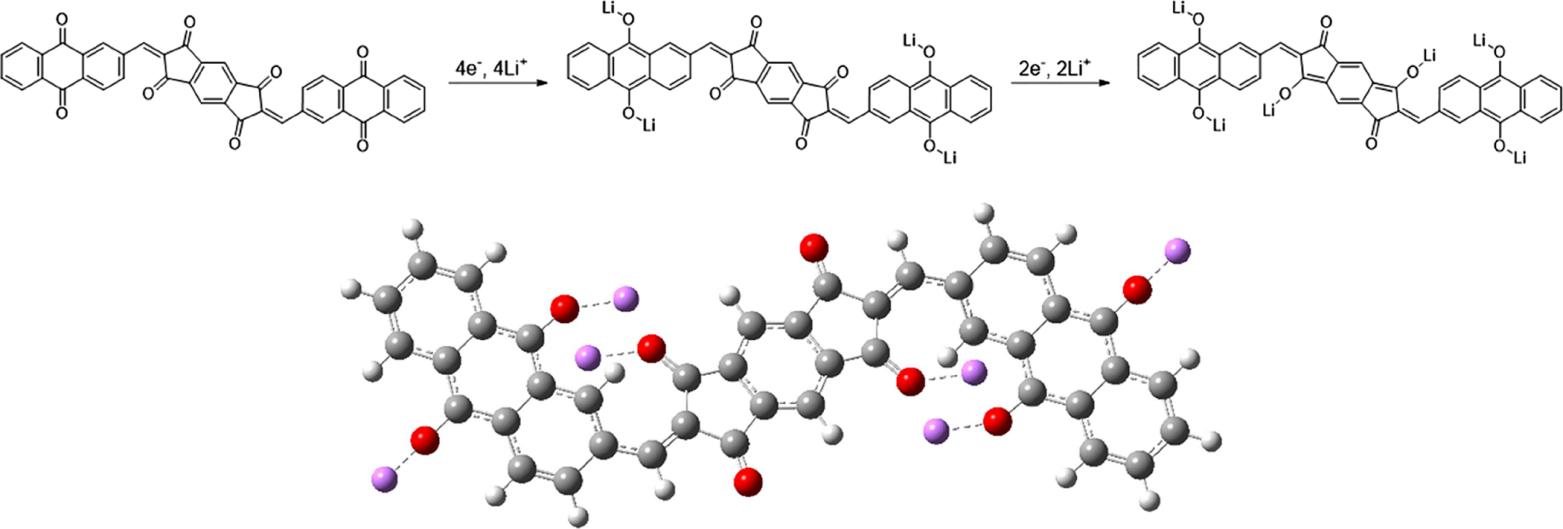
DFT-predicted reduction mechanism for **BAQIT** upon Li^+^ insertion and the DFT-optimized structure of a fully intercalated molecule.

The maximum experimental capacity achieved was approximately 250 mAh g^–1^, which matches well with its theoretical capacity of 247 mAh g^–1^ for a six-electron transfer accompanied by the uptake of six lithium ions. CV (Figure 2) demonstrated the uptake of lithium takes place at two main events, as inferred from the presence of two large redox waves. The symmetry of the molecule can favor concerted multielectron reductions to take place in the form of either two three-electron transfer steps or a four-electron transfer step, followed by a two-electron transfer step or vice versa. The energy of each configuration was calculated using DFT to support the hypothesized Li-ion binding mechanism (Figure S16). The calculations have shown an energy difference of 2.66 eV between both redox mechanisms proposed above, with the mechanism involving an initial binding of 4 Li^+^, followed by subsequent binding of 2 Li^+^ being energetically preferred (Figure 4). This matches well with the observed intensities of the redox waves from CV, with the first reduction peak displaying greater intensity compared to the second reduction peak. The proposed mechanism involves a four-electron reduction centered at the carbonyl moieties on the anthraquinone backbone, followed by a two-electron reduction at the carbonyl moieties of the core unit.

Changes in the morphology of the pristine materials and electrode particles at different states of charge were studied by SEM. Owing to the highly conjugated nature of **BAQIT**, it displays a platelet-like morphology (Figure S12). This morphology appears to breakdown to quasi-spherical particles during the electrode preparation process, and this quasi-spherical morphology is retained across different stages of cycling (Figure S13).

**Figure 5:**
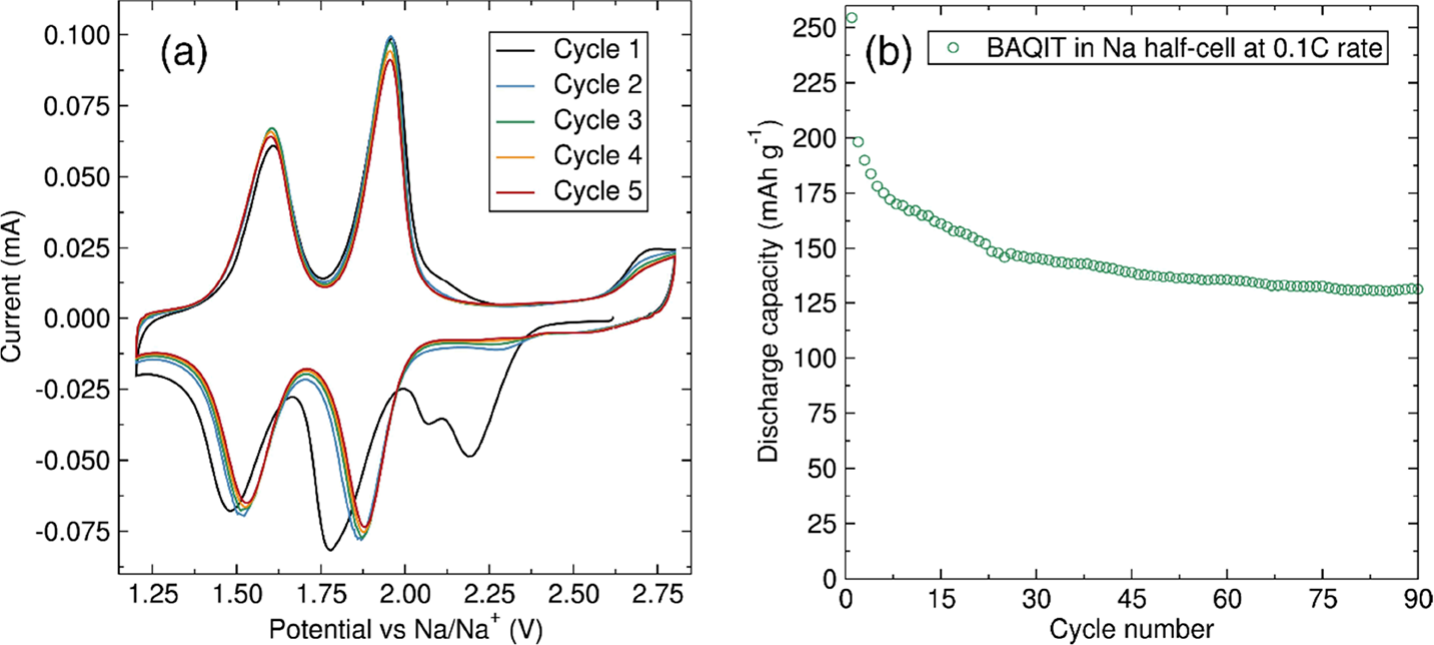
(a) CV at a scan rate at 0.1 mV s^–1^ and (b) discharge capacities observed from galvanostatic cycling at 0.1C rate over 90 cycles of **BAQIT** in Na-ion half-cells.

CV and galvanostatic cycling experiments were also performed in Na-ion half-cells with Na metal as the counter and reference electrode and NaPF_6_ in dioxolane (DOL) plus DME solvent mixture (Figure 5a). The CVs show two distinct redox peaks with similar intensity ratios as those found for Li-ion cells. The voltage of the first reduction peak is centered at 1.9 V, which is ca. 0.3 V lower compared to these of the Li-ion, as expected from the potential difference between Na and Li metal. The second reduction peak is centered just above 1.5 V, which in this case is lower than the Li and Na potential difference. This lower potential can be attributed to the difference in energies between the initially lithiated and sodiated materials after the first redox reaction. The larger size and lower polarization of Na^+^ could result in a lower intercalation potential related to the lower inductive effect of Na^+^ with **BAQIT**. In addition, an irreversible initial reduction redox wave in the first cycle is noted above 2 V, which may suggest the formation of a cathode electrolyte interphase.

**Figure 6:**
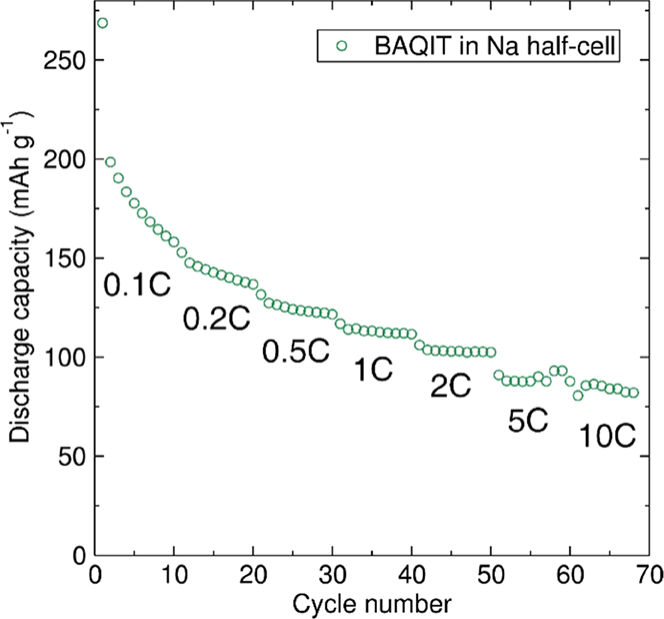
Rate capability of **BAQIT** in a Na metal half-cell cycled at increasing rates.

The galvanostatic cycling profiles for **BAQIT** in Na-ion half-cells are reminiscent of those for Li-ion half-cells, with two distinctive plateaus matching the potential values of the redox peaks observed from CV (Figure S8). Figure 5b shows the evolution of the discharge capacity where an initial discharge capacity near 250 mAh g^–1^ was achieved in the first cycle, which decreases to 200 mAh g^–1^ in the second cycle, which could be attributed to the irreversible formation of the interfacial layer. The capacity then stabilizes, reaching 130 mAh g^–1^ after 90 cycles. Increasing the upper voltage cutoff to 3.2 V for a direct comparison with Li cell galvanostatic cycling results in the appearance of small irreversible redox contributions, leading to lower capacity retention (Figure S9). Hence, a potential window of 1.2 to 2.8 V was maintained for high-rate and long-term cycling tests. Cycling **BAQIT** at 0.5C and 2C in Na half-cells demonstrated discharge capacities of 108.8 and 86 mAh g^–1^ after 200 and 500 cycles, respectively. The rate capability was also evaluated by cycling the material at various specific currents ranging from 0.1C to 10C rate against Na metal (Figure 6). Reversible capacities of 158, 136, 121, 112, 102, and 88 mAh g^–1^ were achieved at current densities of 0.1C, 0.2C, 0.5C, 1C, 2C, and 5C, respectively. Notably, even at a high current of 10C, a reversible capacity of 82 mAh g^–1^ is observed. The device performance of **BAQIT** is comparable with previously described small-molecule sodium-ion battery materials.^57,58^

GITT measurements were carried out to investigate the resulting Na^+^ diffusion properties (Figure S11), and diffusion lengths ranging between 10^–6^ and 10^–4^ s^–1^ were calculated. The symmetry of the molecule favors concerted multielectron reductions in the form of either two three-electron transfer steps or a four-electron transfer step, followed by a two-electron transfer step or vice versa. This is consistent with CV measurements (Figure 5), which indicate two distinct redox events upon charge/discharge. DFT calculations for each possible pathway (Figure S17) reveal a similar reduction process and sodium-binding pathway, as observed earlier for lithium, with a four-electron reduction first and corresponding sodium binding followed by a two-electron process. A small difference of 0.10 eV was found between competing pathways. At full sodium binding, the molecule displays greater planarity compared to the related lithiated molecule (Figure S15). This is likely due to the relative size of the larger Na^+^ ions that are less easily accommodated by the molecule. The smaller Li^+^ ions allow the molecule to twist slightly, allowing the central Li^+^ ions to interact with both the oxygen ions in the core and the anthraquinone units. This affords additional stability toward one lithiation pathway, which is not viable for sodium binding and explains the larger discrepancy in energy between competing lithium pathways compared with related sodium pathways.

## 3 Conclusions

We have designed and synthesized the multivalent **BAQIT** cathode material and have demonstrated its considerable promise as a cathode material for Li- and Na-ion batteries. **BAQIT** displays a stable six-electron transfer reaction with a 190 mAh g^–1^ reversible capacity after 300 cycles at 0.1C rate in Li half-cells. Remarkably, **BAQIT** shows a capacity of 142 mAh g^–1^ even at a 10C rate (6 min discharge) in Li half-cells, indicating its excellent high-power capability, as well as excellent high cycle stability (86% capacity retention at 2C rate after 2200 cycles). This high energy density, power density, and cycling stability of **BAQIT** can be attributed to its extended conjugated structure, which helps improve intermolecular π–π interactions, facilitating charge transport. This results in an improvement in rate performance and decreases the solubility of the electrode material in the electrolyte, hence improving cycle life. DFT calculations together with ex situ Fourier transform infrared (ATR-IR) spectroscopy have confirmed the Li^+^-ion binding mechanism, where the reduction of four anthraquinone carbonyl groups is followed by the reduction of two carbonyl units of the s-indacene-1,3,5,7(2H,6H)-tetraone core moiety. **BAQIT** also offers functionality as a Na-ion battery cathode, achieving an initial specific capacity of over 200 mAh g^–1^ as well as good capacity retention at high cycling rates (82 mAh g^–1^ at 10C). Our work demonstrates that the highly conjugated **BAQIT** structure offers high capacity and excellent rate capability as a cathode material, thereby showing great promise for high-performance sustainable rechargeable batteries.

## Supporting Information Available

Material synthesis and characterization, solubility study, electrode preparation, electrochemical measurements, modeling, synthesis, battery fabrication, and additional battery characterization data (PDF)

## References

[ref1] Borah R.; Hughson F. R.; Johnston J.; Nann T. On Battery Materials and Methods. Mater. Today Adv. 2020, 6, 100046 10.1016/j.mtadv.2019.100046.

[ref2] Chu S.; Cui Y.; Liu N. The Path towards Sustainable Energy. Nat. Mater. 2016, 16, 16–22. 10.1038/nmat4834. 27994253

[ref3] Larcher D.; Tarascon J.-M. Towards Greener and More Sustainable Batteries for Electrical Energy Storage. Nat. Chem. 2015, 7, 19–29. 10.1038/nchem.2085. 25515886

[ref4] Lu Y.; Chen J. Prospects of Organic Electrode Materials for Practical Lithium Batteries. Nat. Rev. Chem. 2020, 4, 127–142. 10.1038/s41570-020-0160-9. 37128020

[ref5] Winter M.; Barnett B.; Xu K. Before Li Ion Batteries. Chem. Rev. 2018, 118, 11433–11456. 10.1021/acs.chemrev.8b00422. 30500179

[ref6] Goodenough J. B. Energy Storage Materials: A Perspective. Energy Storage Mater. 2015, 1, 158–161. 10.1016/j.ensm.2015.07.001.

[ref7] Manzetti S.; Mariasiu F. Electric Vehicle Battery Technologies: From Present State to Future Systems. Renew. Sustain. Energy Rev. 2015, 51, 1004–1012. 10.1016/j.rser.2015.07.010.

[ref8] Li M.; Lu J.; Chen Z.; Amine K. 30 Years of Lithium-Ion Batteries. Adv. Mater. 2018, 30, 1800561 10.1002/adma.201800561. 29904941

[ref9] Nitta N.; Wu F.; Lee J. T.; Yushin G. Li-Ion Battery Materials: Present and Future. Mater. Today 2015, 18, 252–264. 10.1016/j.mattod.2014.10.040.

[ref10] Liang Y.; Tao Z.; Chen J. Organic Electrode Materials for Rechargeable Lithium Batteries. Adv. Energy Mater. 2012, 2, 742–769. 10.1002/aenm.201100795.

[ref11] Li H.; Wang Z.; Chen L.; Huang X. Research on Advanced Materials for Li-Ion Batteries. Adv. Mater. 2009, 21, 4593–4607. 10.1002/adma.200901710.

[ref12] Bhosale M. E.; Chae S.; Kim J. M.; Choi J. Y. Organic Small Molecules and Polymers as an Electrode Material for Rechargeable Lithium Ion Batteries. J. Mater. Chem. A 2018, 19885–19911. 10.1039/c8ta04906h.

[ref13] Manthiram A. A Reflection on Lithium-Ion Battery Cathode Chemistry. Nat. Commun. 2020, 11, 1550 10.1038/s41467-020-15355-0. 32214093 PMC7096394

[ref14] Chen Z.; Zhang W.; Yang Z. A Review on Cathode Materials for Advanced Lithium Ion Batteries: Microstructure Designs and Performance Regulations. Nanotechnology 2019, 31, 12001 10.1088/1361-6528/ab4447. 31519017

[ref15] Kraytsberg A.; Ein-Eli Y. Higher, Stronger, Better... A Review of 5 Volt Cathode Materials for Advanced Lithium-Ion Batteries. Adv. Energy Mater. 2012, 2, 922–939. 10.1002/aenm.201200068.

[ref16] Shanmukaraj D.; Ranque P.; Ben Youcef H.; Rojo T.; Poizot P.; Grugeon S.; Laruelle S.; Guyomard D. Review—Towards Efficient Energy Storage Materials: Lithium Intercalation/Organic Electrodes to Polymer Electrolytes—A Road Map (Tribute to Michel Armand). J. Electrochem. Soc. 2020, 167, 70530 10.1149/1945-7111/ab787a.

[ref17] Cariello M.; Johnston B.; Bhosale M.; Amores M.; Wilson E.; McCarron L. J.; Wilson C.; Corr S. A.; Cooke G. Benzo-Dipteridine Derivatives as Organic Cathodes for Li- and Na-Ion Batteries. ACS Appl. Energy Mater. 2020, 3, 8302–8308. 10.1021/acsaem.0c00829. 33015587 PMC7525807

[ref18] Qin K.; Huang J.; Holguin K.; Luo C. Recent Advances in Developing Organic Electrode Materials for Multivalent Rechargeable Batteries. Energy Environ. Sci. 2020, 13, 3950–3992. 10.1039/d0ee02111c.

[ref19] Gannett C. N.; Melecio-Zambrano L.; Theibault M. J.; Peterson B. M.; Fors B. P.; Abruña H. D. Organic Electrode Materials for Fast-Rate, High-Power Battery Applications. Mater. Rep. Energy 2021, 1, 100008 10.1016/j.matre.2021.01.003.

[ref20] Dunn B.; Kamath H.; Tarascon J.-M. Electrical Energy Storage for the Grid: A Battery of Choices. Science 2011, 334, 928–935. 10.1126/science.1212741. 22096188

[ref21] Yang Z.; Zhang J.; Kintner-Meyer M. C. W.; Lu X.; Choi D.; Lemmon J. P.; Liu J. Electrochemical Energy Storage for Green Grid. Chem. Rev. 2011, 111, 3577–3613. 10.1021/cr100290v. 21375330

[ref22] Luo W.; Allen M.; Raju V.; Ji X. An Organic Pigment as a High-Performance Cathode for Sodium-Ion Batteries. Adv. Energy Mater. 2014, 4, 1400554 10.1002/aenm.201400554.

[ref23] Shi R.; Liu L.; Lu Y.; Wang C.; Li Y.; Li L.; Yan Z.; Chen J. Nitrogen-Rich Covalent Organic Frameworks with Multiple Carbonyls for High-Performance Sodium Batteries. Nat. Commun. 2020, 11, 178 10.1038/s41467-019-13739-5. 31924753 PMC6954217

[ref24] Vitaku E.; Gannett C. N.; Carpenter K. L.; Shen L.; Abruña H. D.; Dichtel W. R. Phenazine-Based Covalent Organic Framework Cathode Materials with High Energy and Power Densities. J. Am. Chem. Soc. 2019, 142, 16–20. 10.1021/jacs.9b08147. 31820958

[ref25] Acker P.; Speer M. E.; Wössner J. S.; Esser B. Azine-Based Polymers with a Two-Electron Redox Process as Cathode Materials for Organic Batteries. J. Mater. Chem. A 2020, 8, 11195–11201. 10.1039/d0ta04083e.

[ref26] Li Q.; Li D.; Wang H.; Wang H.; Li Y.; Si Z.; Duan Q. Conjugated Carbonyl Polymer-Based Flexible Cathode for Superior Lithium-Organic Batteries. ACS Appl. Mater. Interfaces 2019, 11, 28801–28808. 10.1021/acsami.9b06437. 31313916

[ref27] Zhu L. M.; Lei A. W.; Cao Y. L.; Ai X. P.; Yang H. X. An All-Organic Rechargeable Battery Using Bipolar Polyparaphenylene as a Redox-Active Cathode and Anode. Chem. Commun. 2013, 49, 567–569. 10.1039/c2cc36622c. 23212556

[ref28] Gospodinova N.; Terlemezyan L. Conducting Polymers Prepared by Oxidative Polymerization: Polyaniline. Prog. Polym. Sci. 1998, 23, 1443–1484. 10.1016/s0079-6700(98)00008-2.

[ref29] Xie J.; Wang Z.; Xu Z. J.; Zhang Q. Toward a High-Performance All-Plastic Full Battery with a’Single Organic Polymer as Both Cathode and Anode. Adv. Energy Mater. 2018, 8, 1703509 10.1002/aenm.201703509.

[ref30] Deng S.-R.; Kong L.-B.; Hu G.-Q.; Wu T.; Li D.; Zhou Y.-H.; Li Z.-Y. Benzene-Based Polyorganodisulfide Cathode Materials for Secondary Lithium Batteries. Electrochim. Acta 2006, 51, 2589–2593. 10.1016/j.electacta.2005.07.045.

[ref31] Koshika K.; Chikushi N.; Sano N.; Oyaizu K.; Nishide H. A TEMPO-Substituted Polyacrylamide as a New Cathode Material: An Organic Rechargeable Device Composed of Polymer Electrodes and Aqueous Electrolyte. Green Chem. 2010, 12, 1573 10.1039/b926296b.

[ref32] Mauger A.; Julien C.; Paolella A.; Armand M.; Zaghib K. Recent Progress on Organic Electrodes Materials for Rechargeable Batteries and Supercapacitors. Materials 2019, 12, 1770 10.3390/ma12111770. PMC6600696 31159168

[ref33] Kong L.; Liu M.; Huang H.; Xu Y.; Bu X. Metal/Covalent-Organic Framework Based Cathodes for Metal-Ion Batteries. Adv. Energy Mater. 2021, 2100172 10.1002/aenm.202100172.

[ref34] Liang Y.; Zhao C.; Yuan H.; Chen Y.; Zhang W.; Huang J.; Yu D.; Liu Y.; Titirici M.; Chueh Y.; Yu H.; Zhang Q. A Review of Rechargeable Batteries for Portable Electronic Devices. InfoMat 2019, 1, 6–32. 10.1002/inf2.12000.

[ref35] Niebel C.; Lokshin V.; Khodorkovsky V. A General Approach toward Janus Diones: Synthesis of Dicyclopenta[b,g]Naphthalene-1,3,6,8(2H,7H)-Tetraone. Tetrahedron Lett. 2008, 49, 7276–7278. 10.1016/j.tetlet.2008.10.028.

[ref36] Zitzler-Kunkel A.; Lenze M. R.; Schnier T.; Meerholz K.; Würthner F. Comparative Studies on Optical, Redox, and Photovoltaic Properties of a Series of D-A-D and Analogous D-A Chromophores. Adv. Funct. Mater. 2014, 24, 4645–4653. 10.1002/adfm.201400455.

[ref37] Githaiga G. W.; Woodward A. W.; Morales A. R.; Bondar M. V.; Belfield K. D. Photophysical and Computational Analysis of a Symmetrical Fluorene-Based Janus Dione Derivative. J. Phys. Chem. C 2015, 119, 21053–21059. 10.1021/acs.jpcc.5b04840.

[ref38] Akaike K.; Enozawa H.; Kajitani T.; Koizumi M.; Kosaka A.; Hashizume D.; Koizumi Y.; Saeki A.; Seki S.; Fukushima T. Tetrathiafulvalene Hybridized with Indacenetetraone as Visible-Light-Harvesting Electron Acceptor Applicable to Bulk-Heterojunction Organic Photovoltaics. Chem. Lett. 2013, 42, 1417–1419. 10.1246/cl.130702.

[ref40] Bachman J. E.; Curtiss L. A.; Assary R. S. Investigation of the Redox Chemistry of Anthraquinone Derivatives Using Density Functional Theory. J. Phys. Chem. A 2014, 118, 8852–8860. 10.1021/jp5060777. 25159500

[ref41] Takeda T.; Taniki R.; Masuda A.; Honma I.; Akutagawa T. Electron-Deficient Anthraquinone Derivatives as Cathodic Material for Lithium Ion Batteries. J. Power Sources 2016, 328, 228–234. 10.1016/j.jpowsour.2016.08.022.

[ref42] Zhang K.; Guo C.; Zhao Q.; Niu Z.; Chen J. High-Performance Organic Lithium Batteries with an Ether-Based Electrolyte and 9,10-Anthraquinone (AQ)/CMK-3 Cathode. Adv. Sci. 2015, 2, 1500018 10.1002/advs.201500018. PMC5115363 27980937

[ref43] Song Z.; Qian Y.; Gordin M. L.; Tang D.; Xu T.; Otani M.; Zhan H.; Zhou H.; Wang D. Polyanthraquinone as a Reliable Organic Electrode for Stable and Fast Lithium Storage. Angew. Chem., Int. Ed. 2015, 127, 13947–14157. 10.1002/ange.201506673. 26411505

[ref44] Song Z.; Zhan H.; Zhou Y. Anthraquinone based polymer as high performance cathode material for rechargeable lithium batteries. Chem. Commun. 2009, 4, 448–450. 10.1039/B814515F. 19137181

[ref45] Kawai T.; Oyaizu K.; Nishide H. High-Density and Robust Charge Storage with Poly(anthraquinone-substituted norbornene) for Organic Electrode-Active Materials in Polymer–Air Secondary Batteries. Macromolecules 2015, 48, 2429–2434. 10.1021/ma502396r.

[ref46] Yao M.; Yamazaki S.-I.; Senoh H.; Sakai T.; Kiyobayashi T. Crystalline polycyclic quinone derivatives as organic positive-electrode materials for use in rechargeable lithium batteries. Mater. Sci. Eng., B 2012, 177, 483–487. 10.1016/j.mseb.2012.02.007.

[ref47] Yang J.; Su H.; Wang Z.; Sun P.; Xu Y. An Insoluble Anthraquinone Dimer with Near-Plane Structure as a Cathode Material for Lithium-Ion Batteries. ChemSusChem 2020, 13, 2436–2442. 10.1002/cssc.201903227. 31840438

[ref39] Walker W.; Grugeon S.; Mentre O.; Laruelle S.; Tarascon J.-M.; Wudl F. Ethoxycarbonyl-Based Organic Electrode for Li-Batteries. J. Am. Chem. Soc. 2010, 132, 6517–6523. 10.1021/ja1012849. 20405915

[ref48] Li W.; Lee S.; Manthiram A. High-Nickel NMA: A Cobalt-Free Alternative to NMC and NCA Cathodes for Lithium-Ion Batteries. Adv. Mater. 2020, 2002718 10.1002/adma.202002718. 32627875

[ref49] Xie J.; Chen W.; Long G.; Gao W.; Xu Z. J.; Liu M.; Zhang Q. Boosting the Performance of Organic Cathodes through Structure Tuning. J. Mater. Chem. A 2018, 6, 12985–12991. 10.1039/c8ta03857k.

[ref50] Tian R.; Park S.-H.; King P. J.; Cunningham G.; Coelho J.; Nicolosi V.; Coleman J. N. Quantifying the Factors Limiting Rate Performance in Battery Electrodes. Nat. Commun. 2019, 10, 1933 10.1038/s41467-019-09792-9. 31036866 PMC6488605

[ref51] Kwon J. E.; Hyun C.-S.; Ryu Y. J.; Lee J.; Min D. J.; Park M. J.; An B.-K.; Park S. Y. Triptycene-Based Quinone Molecules Showing Multi-Electron Redox Reactions for Large Capacity and High Energy Organic Cathode Materials in Li-Ion Batteries. J. Mater. Chem. A 2018, 6, 3134–3140. 10.1039/c7ta09968a.

[ref52] Yokoji T.; Kameyama Y.; Maruyama N.; Matsubara H. High-Capacity Organic Cathode Active Materials of 2,2′-Bis-p-Benzoquinone Derivatives for Rechargeable Batteries. J. Mater. Chem. A 2016, 4, 5457–5466. 10.1039/c5ta10713j.

[ref53] Yang J.; Xiong P.; Shi Y.; Sun P.; Wang Z.; Chen Z.; Xu Y. Rational Molecular Design of Benzoquinone-Derived Cathode Materials for High-Performance Lithium-Ion Batteries. Adv. Funct. Mater. 2020, 30, 1909597 10.1002/adfm.201909597.

[ref54] An S. Y.; Schon T. B.; Seferos D. S. Stable, Dual Redox Unit Organic Electrodes. ACS Omega 2020, 5, 1134–1141. 10.1021/acsomega.9b03355. 31984270 PMC6977105

[ref55] Assat G.; Delacourt C.; Corte D. A. D.; Tarascon J.-M. Editors’ Choice—Practical Assessment of Anionic Redox in Li-Rich Layered Oxide Cathodes: A Mixed Blessing for High Energy Li-Ion Batteries. J. Electrochem. Soc. 2016, 163, A2965–A2976. 10.1149/2.0531614jes.

[ref56] Griffith K. J.; Wiaderek K. M.; Cibin G.; Marbella L. E.; Grey C. P. Niobium Tungsten Oxides for High-Rate Lithium-Ion Energy Storage. Nature 2018, 559, 556–563. 10.1038/s41586-018-0347-0. 30046074

[ref57] Yin X.; Sarkar S.; Shi S.; Huang Q.-A.; Zhao H.; Yan L.; Zhao Y.; Zhang J. Recent Progress in Advanced Organic Electrode Materials for Sodium-Ion Batteries: Synthesis, Mechanisms, Challenges and Perspectives. Adv. Funct. Mater. 2020, 30, 1908445 10.1002/adfm.201908445.

[ref58] Xu Y.; Zhou M.; Lei Y. Organic materials for rechargeable sodium-ion batteries. Mater. Today 2018, 21, 60–78. 10.1016/j.mattod.2017.07.005.

