# Supplementary material for: A Quinone-Based Cathode Material for High-Performance Organic Lithium and Sodium Batteries
Source: ACS Appl Energy Mater. 2021 Oct 18;4(11):12084–90. doi: 10.1021/acsaem.1c01339 (PMC8611644; doi:10.1021/acsaem.1c01339)
Supplement: Supplementary file 1 — ae1c01339_si_001.pdf [file ae1c01339_si_001.pdf]

Supporting Information

A quinone-based cathode material for high-performance organic lithium and sodium batteries.

Dylan Wilkinson,[a‡] Manik Bhosale,[b‡] Marco Amores,[b‡] Gollapally Naresh,[b] Serena A. Cussen[b,c*] and Graeme Cooke[a,*]

a. School of Chemistry, University of Glasgow, Glasgow G12 8QQ, United Kingdom.
b. Department of Chemical and Biological Engineering, University of Sheffield, Sheffield S1 3JD, United Kingdom.
c. Department of Materials Science and Engineering, University of Sheffield, Sheffield S1 3JD, United Kingdom

Table of Contents:

1. Material synthesis and Characterisation S2
2. Solubility study S4
3. Electrode preparation and electrochemical measurements S5
4. Modelling S13
5. References S16

1. Material synthesis and Characterisation

All starting materials and reagents were purchased from commercial suppliers (Aldrich, TCI or FluoroChem) and used without further purification. Anhydrous solvents were obtained either from Innovative Technology inc. Pure Solv 400-5-MD solvent purification system or from Sigma Aldrich. Mass spectrometry was obtained from the mass spectrometry service at the University of Glasgow or the EPSRC UK National Mass Spectrometry Facility at Swansea University. NMRs were recorded on either Bruker Avance III 400 or Bruker Avance III 500 spectrometers. The ^{1}H spectra were recorded at 400 or 500 MHz, with TMS as internal standard. NMR spectra were analysed using Mestrenova 12.0 Lite Edition. s-Indacene-1,3,5,7(2H,6H)-tetraone[1] and 9,10-Anthraquinone-2-carbaldehyde[2] were synthesised according to literature protocols.

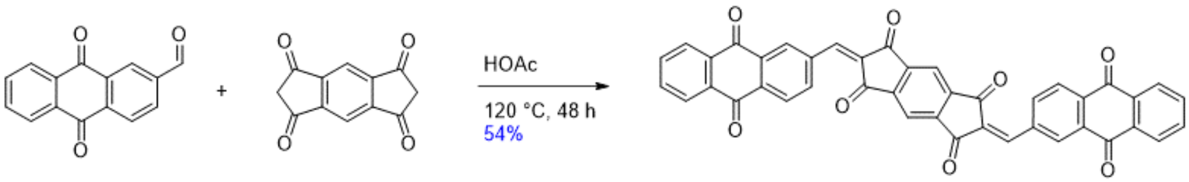

Scheme 1: Synthesis of **BAQIT**.

Synthesis of BAQIT:

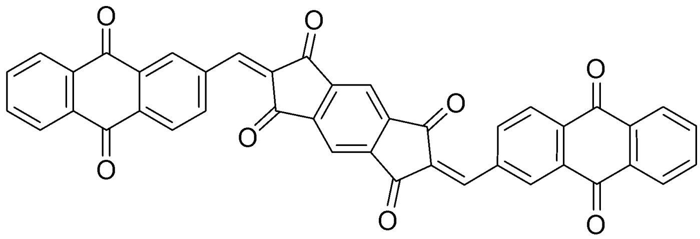

s-Indacene-1,3,5,7(2H,6H)-tetraone (21 mg, 0.1 mmol) and 9,10-anthraquinone-2-carbaldehyde (50 mg, 0.21 mmol) were added to glacial acetic acid (5 mL) and heated under reflux at 110 °C for 24 h. After cooling to room temperature, the mixture was added to water (20 mL) and the precipitate was filtered, washed with water and dried in a vacuum oven. The compound was purified via Soxhlet extractions in dichloromethane, toluene, tetrahydrofuran and methanol yielding **BAQIT** as a brown solid (35 mg, 54%). Poor solubility precluded acquisition of suitable NMR spectra. MS (ASAP^{+}) m/z = 651.1077 [M+H]$^{+}$ (calculated for $C_{42}H_{18}O_{8}^{+}$: 651.1080). Elemental analysis observed: C = 76.34%, H = 2.71%, O = 20.95% (theoretical C = 77.54%, H = 2.79, O = 19.67%). IR: ν_{max}/cm^{-1} = 3082 *w* (C-H), 3030 *w* (C-H), 2949 *w* (C-H), 2909 *w* (C-H), 1747 *m* (C=O), 1709 *s* (C=O), 1684 *s* (C=O), 1667 *s* (C=O), 1613 *m* (C=C), 1587 *m* (C=C arom.), 1560 *w* (C=C arom.), 1447 *w*, 1429 *w*, 1356 *m*, 1330 *m*, 1292 *m*, 1184 *s*, 970 *m*, 931 *m*, 781 *m*, 714 *s*.

Characterisation:

Mass Spectrum

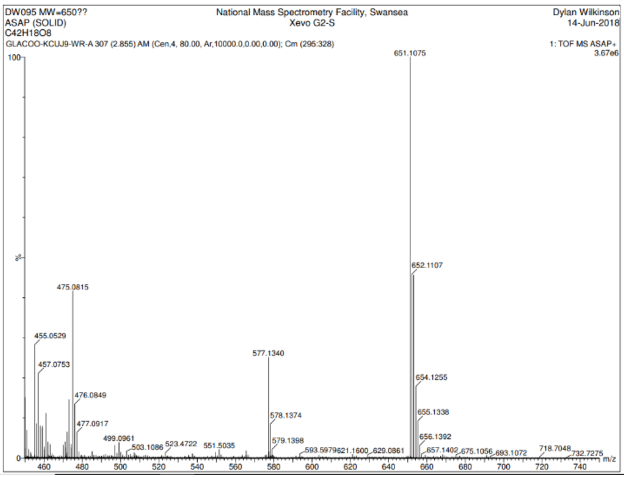

Single Mass Analysis
Tolerance = 5.0 PPM / DBE: min = -50.0, max = 100.0
Element prediction: Off
Number of isotope peaks used for i-FIT = 4

Monoisotopic Mass, Odd and Even Electron Ions
1446 formula(e) evaluated with 16 results within limits (up to 500 closest results for each mass)
Elements Used:
C: 0-60 H: 0-80 N: 0-10 O: 0-18

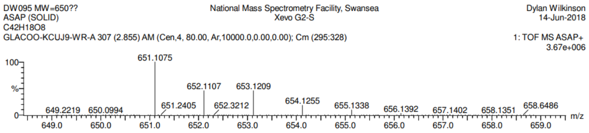

Minimum:				-50.0				
Maximum:		5.0	5.0	100.0				

Mass	Calc. Mass	mDa	PPM	DBE	i-FIT	Norm	Conf(%)	Formula
651.1075	651.1072	0.3	0.5	16.0	724.7	3.524	2.95	C27 H25 N O18
	651.1072	0.3	0.5	21.5	725.0	3.811	2.21	C26 H19 N8 O13
	651.1080	-0.5	-0.8	33.5	723.5	2.291	10.12	C42 H19 O8
	651.1080	-0.5	-0.8	39.0	723.8	2.601	7.42	C41 H13 N7 O3
	651.1066	0.9	1.4	34.0	723.8	2.625	7.25	C40 H17 N3 O7
	651.1066	0.9	1.4	39.5	724.2	2.941	5.28	C39 H11 N10 O2
	651.1085	-1.0	-1.5	21.0	724.7	3.467	3.12	C28 H21 N5 O14
	651.1058	1.7	2.6	16.5	725.1	3.878	2.07	C25 H23 N4 O17
	651.1093	-1.8	-2.8	38.5	723.5	2.283	10.20	C43 H15 N4 O4
	651.1053	2.2	3.4	34.5	724.2	2.978	5.09	C38 H15 N6 O6
	651.1098	-2.3	-3.5	26.0	724.7	3.459	3.15	C29 H17 N9 O10
	651.1098	-2.3	-3.5	20.5	724.4	3.180	4.16	C30 H23 N2 O15
	651.1048	2.7	4.1	47.0	723.3	2.044	12.95	C52 H13 N
	651.1045	3.0	4.6	17.0	725.5	4.232	1.45	C23 H21 N7 O16
	651.1107	-3.2	-4.9	38.0	723.3	2.039	13.01	C45 H17 N O5
	651.1107	-3.2	-4.9	43.5	723.6	2.345	9.59	C44 H11 N8

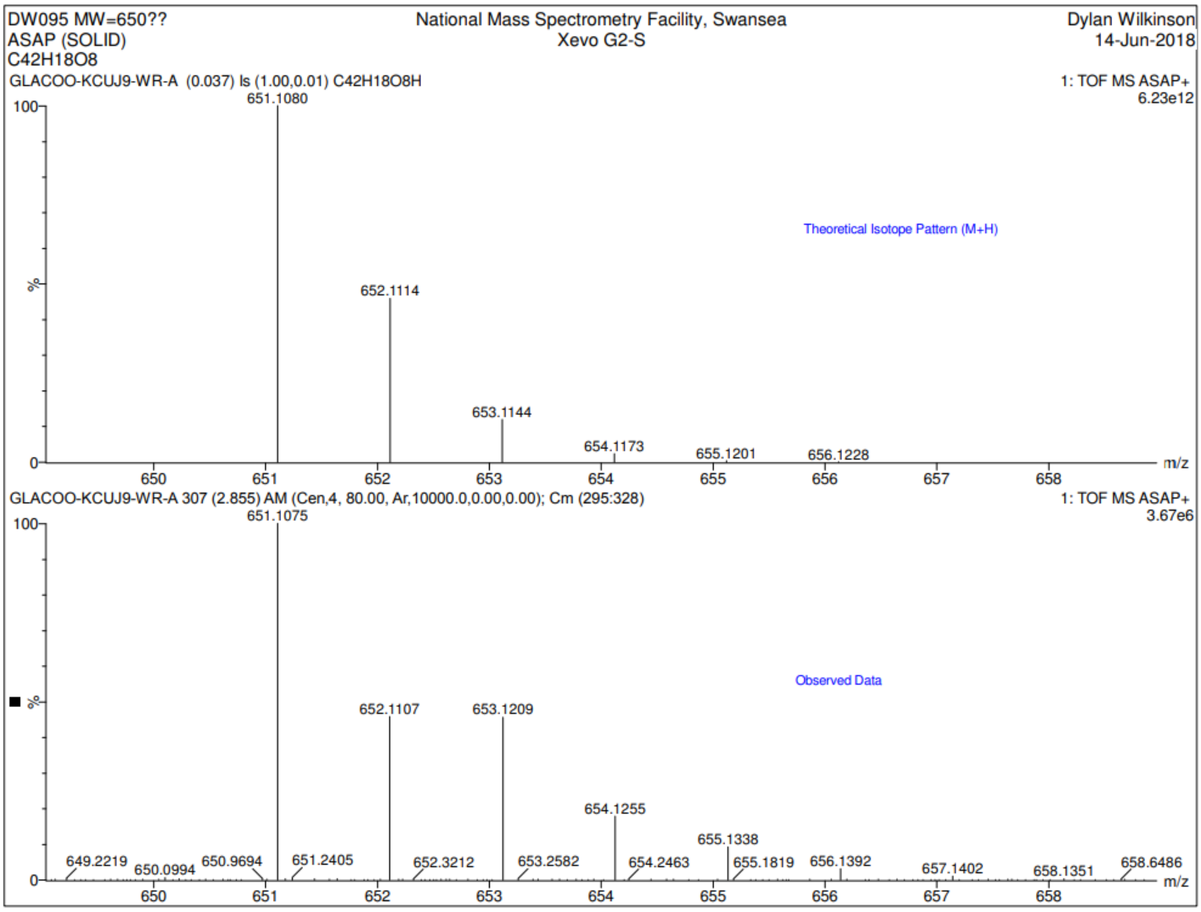

IR Spectrum

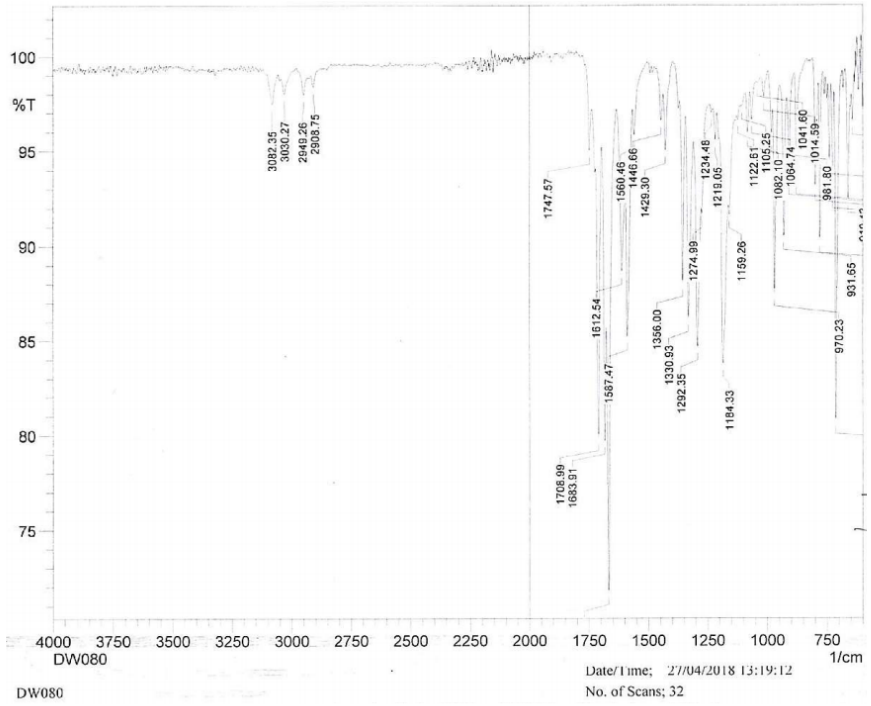

2. ***Solubility Study***

UV-vis spectral measurements were carried out on a Varian *Cary 50 Probe* UV Visible Spectrophotometer. For this, a fixed quantity of AQ, IT and BAQIT (10 mg) were separately taken in 4 mL of DME and a mixture of EC and DME (1:1 v/v ratio). BAQIT was less soluble and settled at the bottom of the vials as compare to the other two molecules shown in the figure S1a and b. To further confirm their solubility in electrolyte, AQ, IT and BAQIT electrode discs were separately taken in 5 mL of electrolyte (with 1,3-dioxolane/DME as reference) and soaked for 2h and 48h before measuring the UV-vis spectra. As shown in figure S1c, the electrolyte in the IT electrode turned to light yellow color after 2h and 48h, respectively. On the other hand, BAQIT exhibited no color or very light color after 2h and 48h soaking and indicates poorer solubility of BAQIT in the electrolyte. The AQ electrode in the electrolyte does not show any color but it showed sharp absorption peaks in the UV-vis spectra along with the IT electrolyte indicating a good solubility of AQ and IT in electrolyte as shown in figure S1 (e) and (f). In contrast BAQIT showed low intensity absorption peaks further confirming its poor solubility.

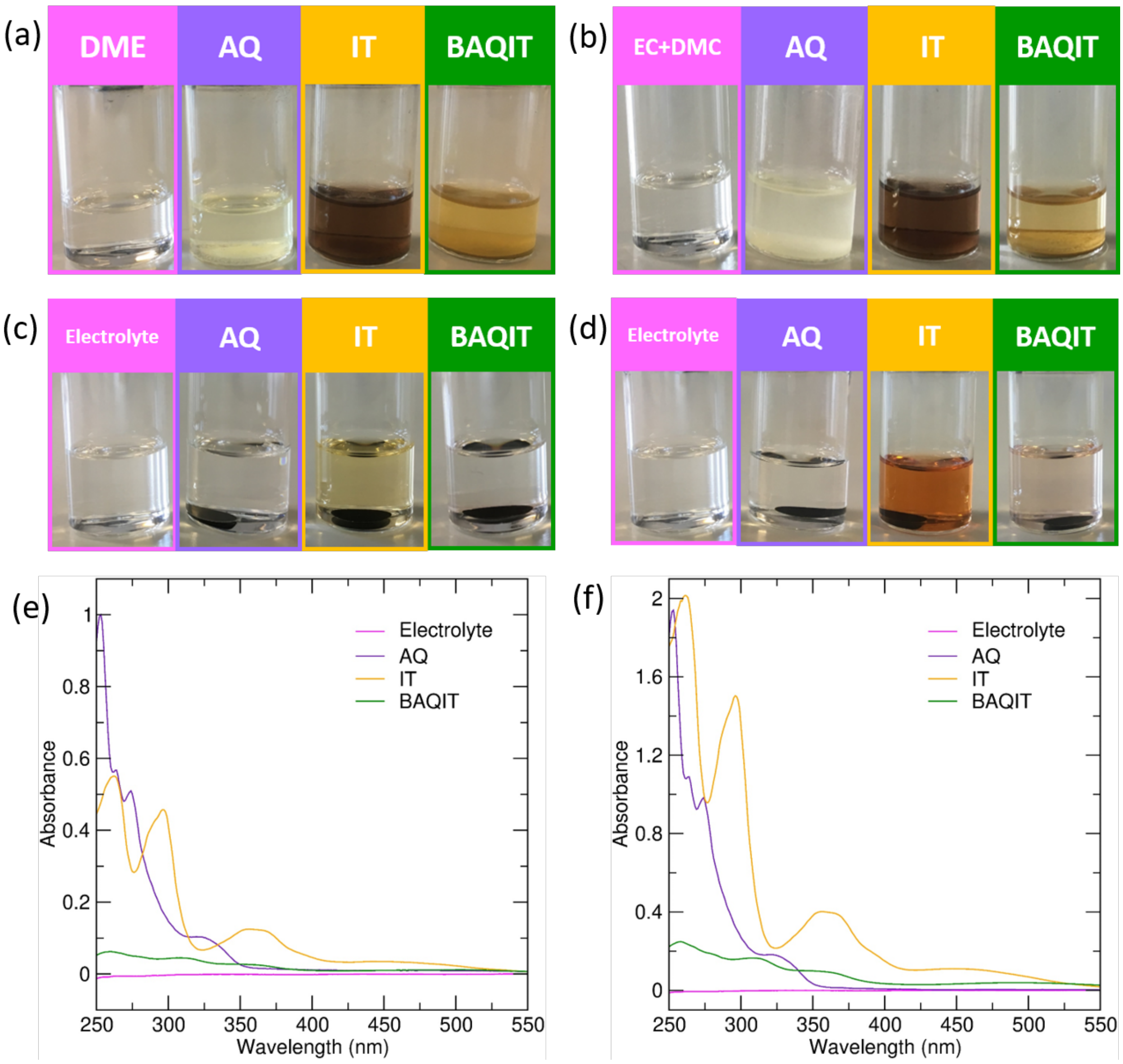

Figure S1 (a) Photographs of 4mL pristine DME, and 10 mg of AQ, IT and BAQIT in 4mL DME, respectively (b) Photographs of 4mL pristine EC with DMC, and 10 mg of AQ, IT and BAQIT in 4mL EC with DMC (1:1 v/v ratio), respectively (c) photographs of the pristine electrolyte, and AQ, IT and BAQIT electrodes separately soaked in 5mL electrolyte (with 1,3-dioxolane/DME as reference) for (c) 2h, (d) 48h. UV-vis spectra of pristine and soaked electrolyte after (e) 2h and (f) 48h.

3. ***Electrode preparation and electrochemical measurements***

All the electrochemical measurements were conducted in CR 2032-type coin cells, assembled in an Ar-filled glovebox. The cathode was composed of a mixture of 60%$_{wt}$ active material, 30%$_{wt}$ Ketjen black and 10%$_{wt}$ polyvinylidene fluoride (PVdF) as binder. The mixture was suspended in N-methyl-2-pyrrolidinone (NMP) to form a slurry, which was coated onto a carbon-coated aluminium foil using a doctor blade. The electrode was dried under vacuum at 80 °C overnight. Coin cells for lithium-ion batteries were assembled using lithium metal as the counter electrode, 1 M LiTFSI in a mixture of DOL/DME (1:1 by volume) as electrolyte and Whatman GF/F glass fibre as the separator. Sodium-ion batteries were assembled using sodium metal as counter electrode, 1 M $LiPF_6$ in a mixture of DOL/DME (1:1 by volume) as electrolyte and Whatman GF/F glass fibre used as the separator. Cyclic voltammetry (CV)

experiments were performed on a VSP-300 Biologic instrument, galvanostatic cycling at different current rates was performed on a MACCOR 4000M Battery and Cell test system and electrochemical impedance spectroscopy (EIS) measurements were carried out on a Solartron ModuLab XM applying a signal amplitude of 5mV in the frequency range of 40 kHz to 10 mHz. Galvanostatic intermittent titration technique (GITT) experiments were carried out by applying 1 h galvanostatic current pulses at a C/10 rate followed by a relaxation period of 5 h at which the voltage evolution was recorded.

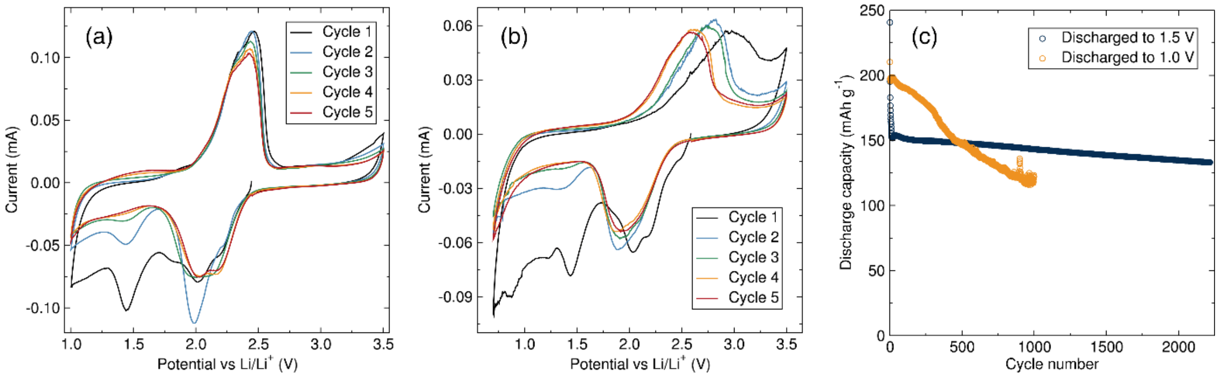

Figure S2: Cyclic voltammetry of **BAQIT** in Li half-cells in the **(a)** 1.0 V to 3.5 V and **(b)** 0.7V to 3.5 V voltage windows. **(c)** Discharge capacity of **BAQIT** in Li half-cell in the 1.5 V to 3.5 V (grey markers) and 1.0 V to 3.5 V (red markers) voltage windows vs Li/Li^+ at cycling rate of 2C.

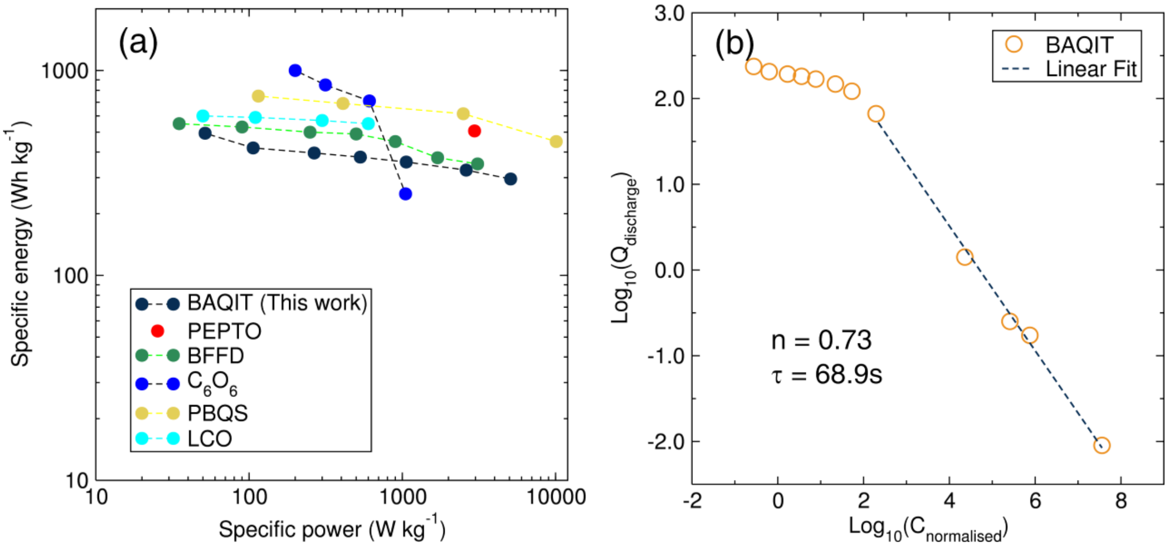

Figure S3: (a) Ragone plot of BAQIT in Li half-cells at room temperature together with best quinone-type power capability materials [poly(2,7-ethynylpyrene-4,5,9,10-tetraone) (PEPTO),[3] benzofuro[5,6-b]furan-4,8-dione (BFFD),[4] C_6O_6,[5] poly(benzoquinonyl sulfide) (PBQS),[6] and high-power modified $LiCoO_2$ (LCO) conventional cathode material.[7] (b) Specific discharge capacity versus normalised delivered capacity, for extracting *τ* (characteristic time associated with charge/discharge) and *n* (exponent of power law decay of capacity).

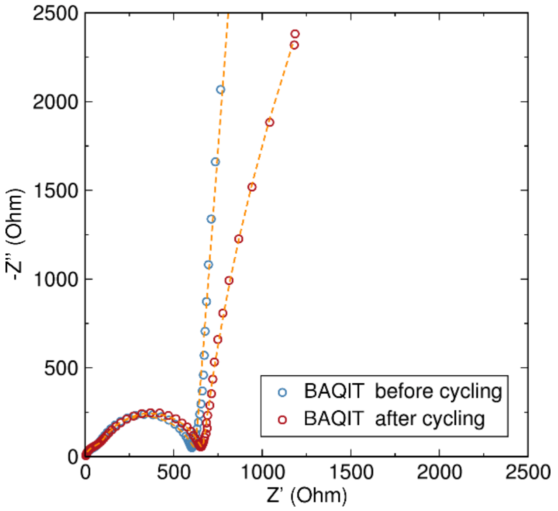

	BAQIT **(Pre)**	BAQIT **(Post)**
R1	3.04	3.02
R2	64.58	78.31
Q2 / a	2.97E-6 / 0.90	3.44 E-6 / 0.90
R3	550.10	581.6
Q3 / a	11.01E-6 / 0.89	13.46 E-6 / 0.88
R4	-	22.85
Q4 / a	4.24E-3 / 0.95	5.40E-3 / 0.93
R5		
Q5 / a		

Figure S4: Nyquist plot from *in-situ* EIS measurements of **BAQIT** in Li half-cell after cell assembly (blue circles) and after ten charge-discharge cycles at 0.1C cycling rate (red circles). Table: Fitted parameter from an equivalent electrical circuit to the Nyquist plot before (pre) and after (post) cycling of **BAQIT** for 10 cycles.

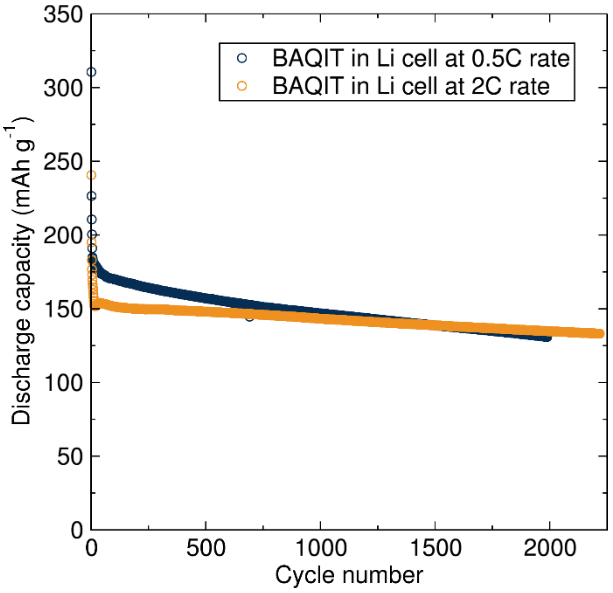

Figure S5: Discharge capacity of **BAQIT** in Li half-cell in the 1.5 V to 3.5 V voltage window vs Li/Li^+ at cycling rates of 0.5C (blue circles) and 2C (orange circles).

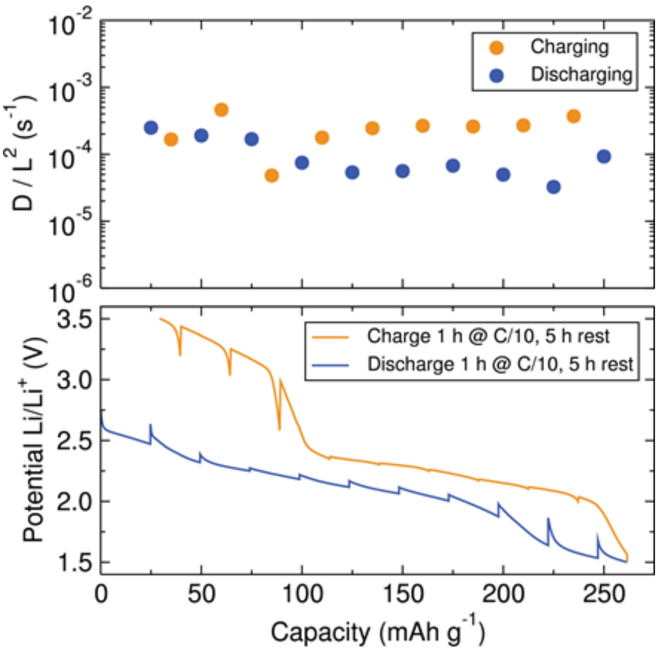

Figure S6: GITT test and diffusion lengths calculated for **BAQIT** cathode material in a Li half-cell cycled at 0.1C for 1 h pulse with a 5 h relaxation period between pulses.

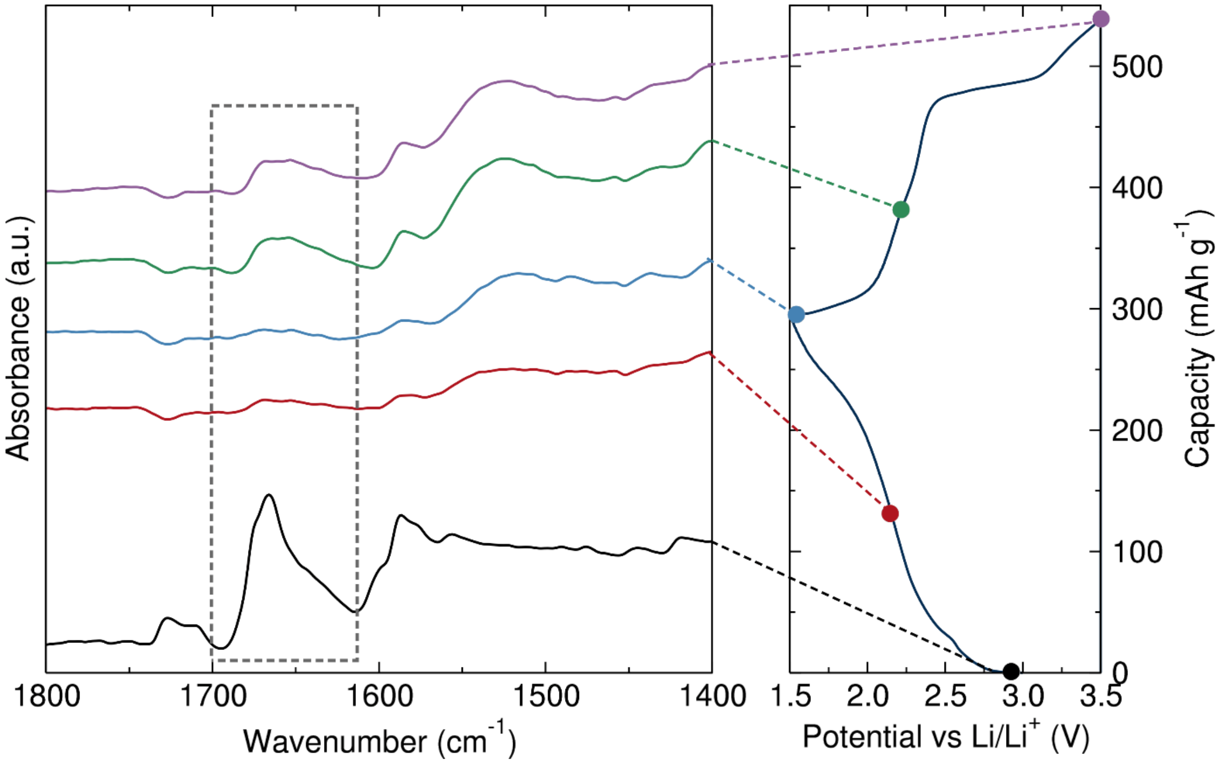

Figure S7: *Ex-situ* FTIR measurements of cycled electrodes to different (dis)charge points.

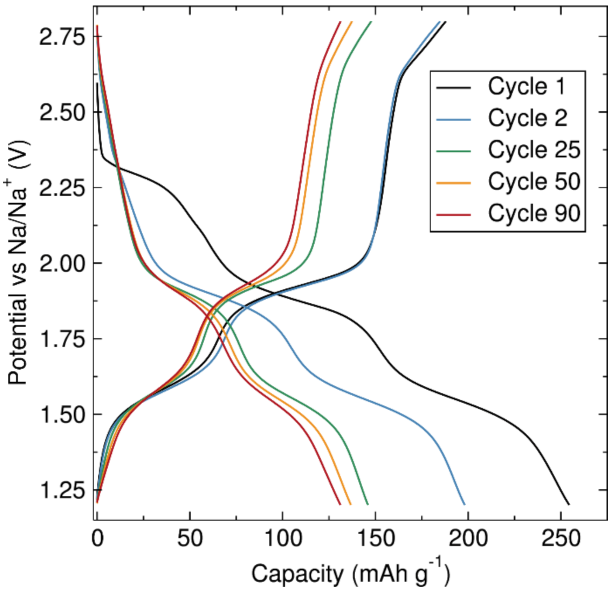

Figure S8: Galvanostatic cycling profile of **BAQIT** in a Na half-cell in the 1.2 V to 2.8 V voltage window vs Na/Na^+ at a cycling rate of 0.1C.

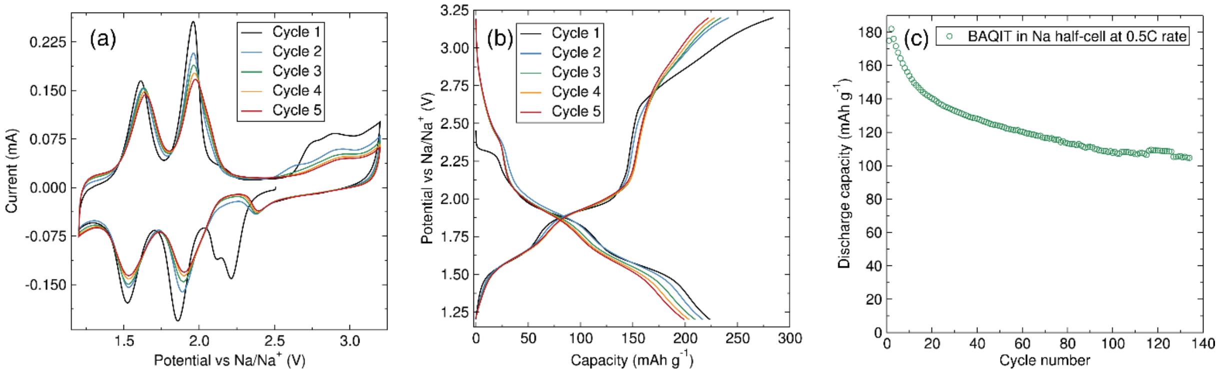

Figure S9 **(a)** CV, **(b)** galvanostatic cycling and **(c)** discharge capacity with prolonged cycling of **BAQIT** in Na half-cell in the 1.2 V to 3.2 V voltage window vs Na/Na^+ at cycling rate of 0.1C.

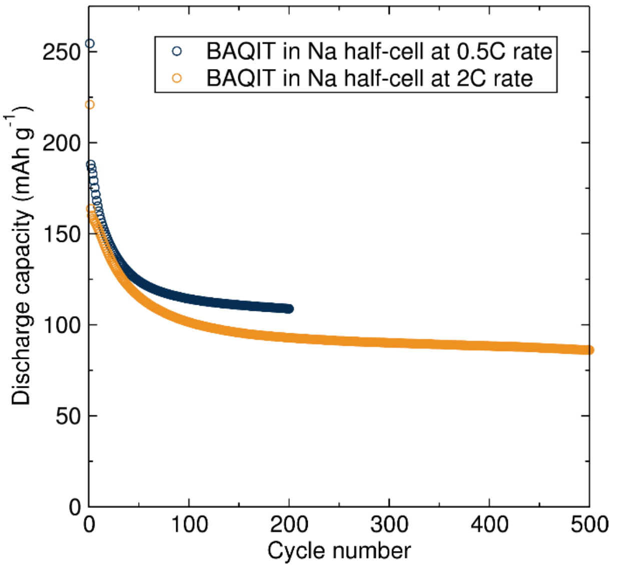

Figure S10: Discharge capacity of **BAQIT** in a Na half-cell in the 1.2 V to 2.8 V voltage window vs Na/Na^+ at cycling rates of 0.5C (blue circles) and 2C (yellow circles).

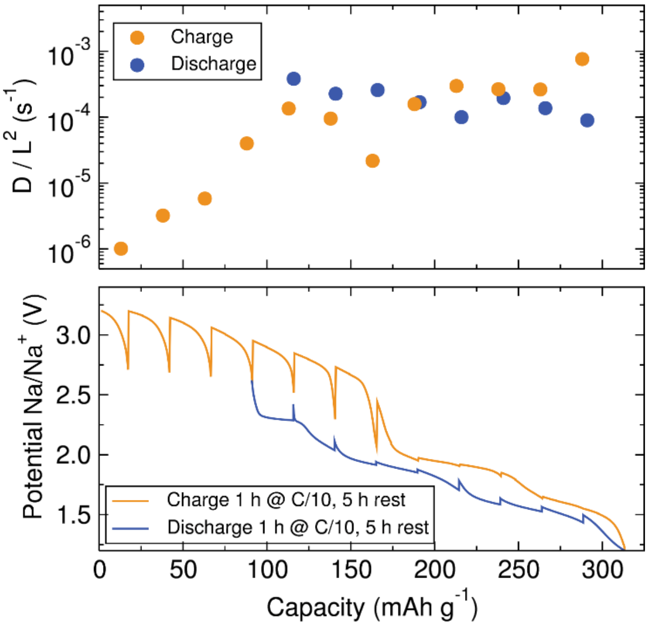

Figure S11: GITT test and diffusion lengths calculated for **BAQIT** in a Na half-cell cycled at 0.1C for 1 h pulse with a 5 h relaxation period between pulses.

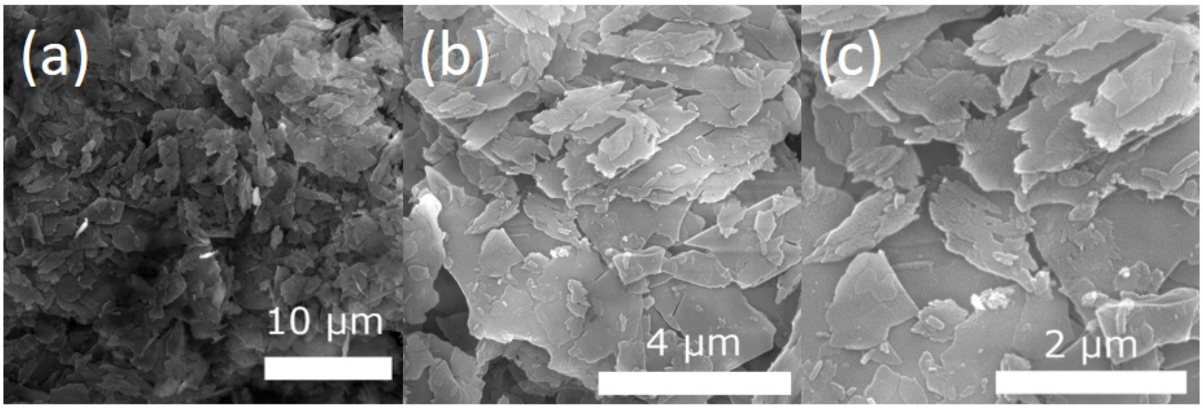

Figure S12: SEM images of pristine **BAQIT**, showing platelet-like morphology.

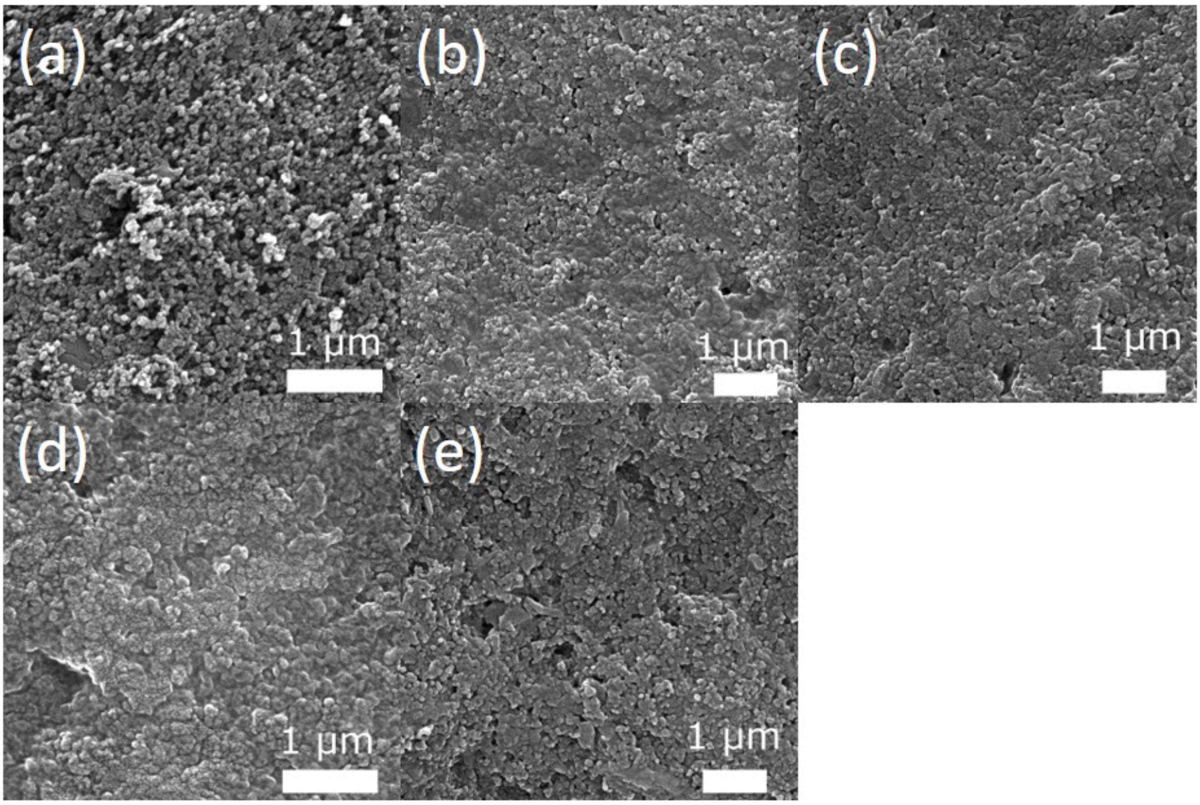

Figure S13: SEM images of **BAQIT** in an **(a)** as-prepared electrode and at different stages of cycling **(b)** half discharge, **(c)** full discharge, **(d)** half charge, **(e)** full charge.

Structure	Theoretical capacity (mAh g^{-1})	Initial reversible capacity (mAh g^{-1}), current density mA g^{-1})	Electrode composition	Electrolyte	Capacity retention (mAh g^{-1}), cycle number, current density (mA g^{-1})	References
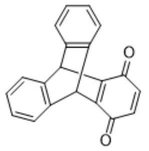	189	122, 0.1C	60:30:10 AM:CB:PVDF	2M LiTFSI 1:1 DME:DOL 1 wt.% $LiNO_3$	61, 20, 0.1C	J. Mater. Chem. A 2018, 6, 3134−3140
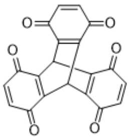	467	387, 0.1C	60:30:10 AM:CB:PVDF	2M LiTFSI 1:1 DME:DOL 1 wt.% $LiNO_3$	213, 20, 0.1C	J. Mater. Chem. A 2018, 6, 3134−3140
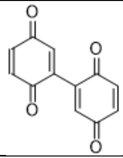	501	380, 40 mA g^{-1}	5:85:10 AM:VGCF: PTFE	2.75M LiTFSI in tetraglyme	198, 50, n.d.	J. Mater. Chem. A 2016, 4, 5457-5466
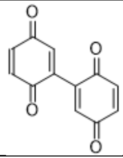	501	300, 0.1C	60:30:10 AM:Graphene: PVDF	2M LiTFSI 1:1 DOL:DME	149, 100, 0.1C	Adv. Funct. Mater. 2020, 1909597
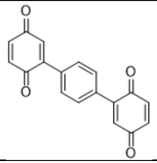	370	367, 0.1C	60:30:10 AM:Graphene: PVDF	2M LiTFSI 1:1 DOL:DME	306, 100, 0.1C	Adv. Funct. Mater. 2020, 1909597
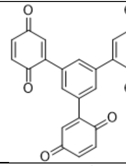	406	397, 0.1C	60:30:10 AM:Graphene: PVDF	2M LiTFSI 1:1 DOL:DME	217, 100, 0.1C	Adv. Funct. Mater. 2020, 1909597
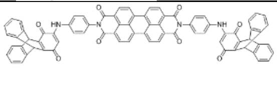	141	88, 0.1C	30:60:10 AM:Super P:PVDF	1M LiPF6 1:9 EC:DME	88%, 200, 1C; 77% 500, 1C	ACS Omega 2020, 5, 2, 1134–1141
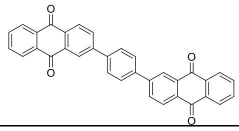	218	212, 0.2C	60:30:10 AM:CNT:SA	2M LiTFSI 1:1 DOL:DME	92%, 100, 0.2C	ChemSusChem 2020, 13 (9), 2436-2442.
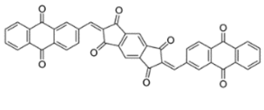	**247**	**295, 0.1C**	**60:30:10 AM:Ketjen black:PVDF**	**1M LiTFSI 1:1 DOL:DME**	**190, 300, 0.1C 130, 1990, 0.5C 130, 2200, 2C**	**This work**

Table S1: Comparison of a selection of different dione-containing small molecule organic cathode performance in Li-ion batteries.

4. Modelling

All calculations were performed at a density functional theory (DFT) level, using Gaussian 09 quantum chemistry suite with Becke-Lee-Yang-Parr (B3LYP) hybrid exchange-correlation functional and 6-311G (2df,2p) basis set.[8] First, the fully oxidised **BAQIT** was geometrically optimised and following this, a frequency optimisation was performed to verify the absence of negative vibrations. The molecule exhibits a planar geometry. (Figure S14).

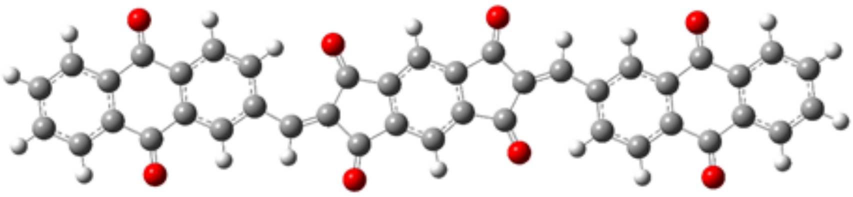

Figure S14. Molecular structure of **BAQIT** calculated by DFT geometry optimisation.

The geometry was then optimised in the presence of lithium. This was done by placing lithium atoms at approximately 4 Å distance from the most likely binding sites resulting in the rearrangement of the system to the most energetically stable configurations. From the electrochemical data, it was evident the molecule undergoes two distinct redox events, and because the capacity equates to an overall 6-electron reduction, it is most likely the molecule undergoes either (i) two 3-electron processes, or (ii) a 4-electron process followed by a 2-electron process. Each possible configuration was optimised, and the energy compared (Figure S16). The same procedure as above was performed in the presence of sodium (Figure S17). It was found that the latter route (ii) was the most energetically favourable for both lithium and sodium intercalation.

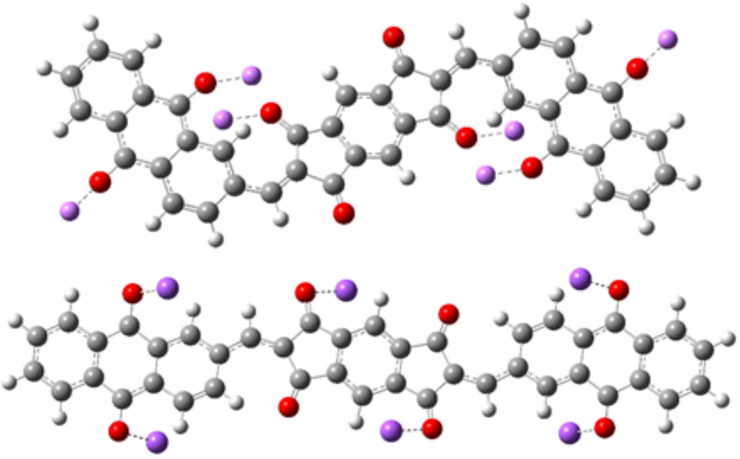

Figure S15. Molecular structure of the most stable conformation of **BAQIT-Li$_6$** and **BAQIT-Na$_6$** calculated by DFT geometry optimisation.

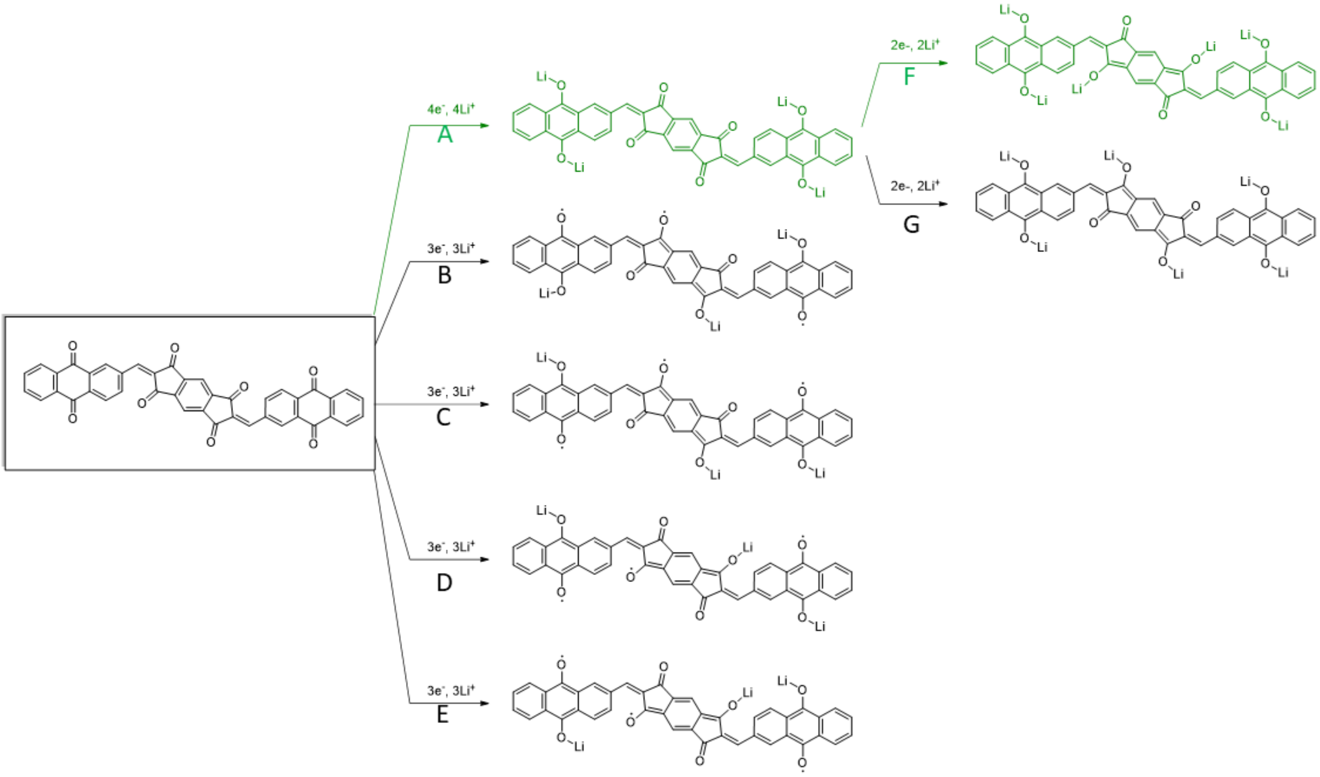

Pathway	Reduction	Resulting Energy (Hartree a.u.)	Optimised Structure
A	$4e^-$, $4Li^+$	-2243.718366	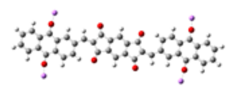
B	$3e^-$, $3Li^+$	-2236.129674	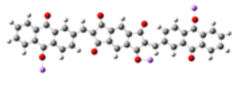
C	$3e^-$, $3Li^+$	-2236.112345	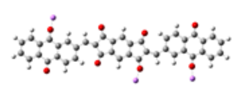
D	$3e^-$, $3Li^+$	-2236.134662	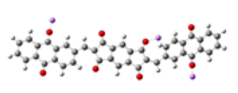
E	$3e^-$, $3Li^+$	-2236.147420	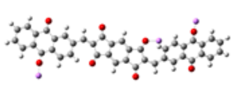
F	$2e^-$, $2Li^+$	-2258.932949	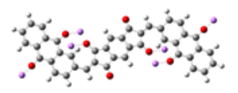
G	$2e^-$, $2Li^+$	-2258.835359	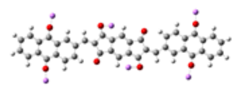

Figure S16: Comparison of reduction/lithium binding pathways of **BAQIT**. Sum of electronic and thermal free energies calculated *via* DFT calculations. The route highlighted in green displays lowest energy and theorized lithium binding mechanism.

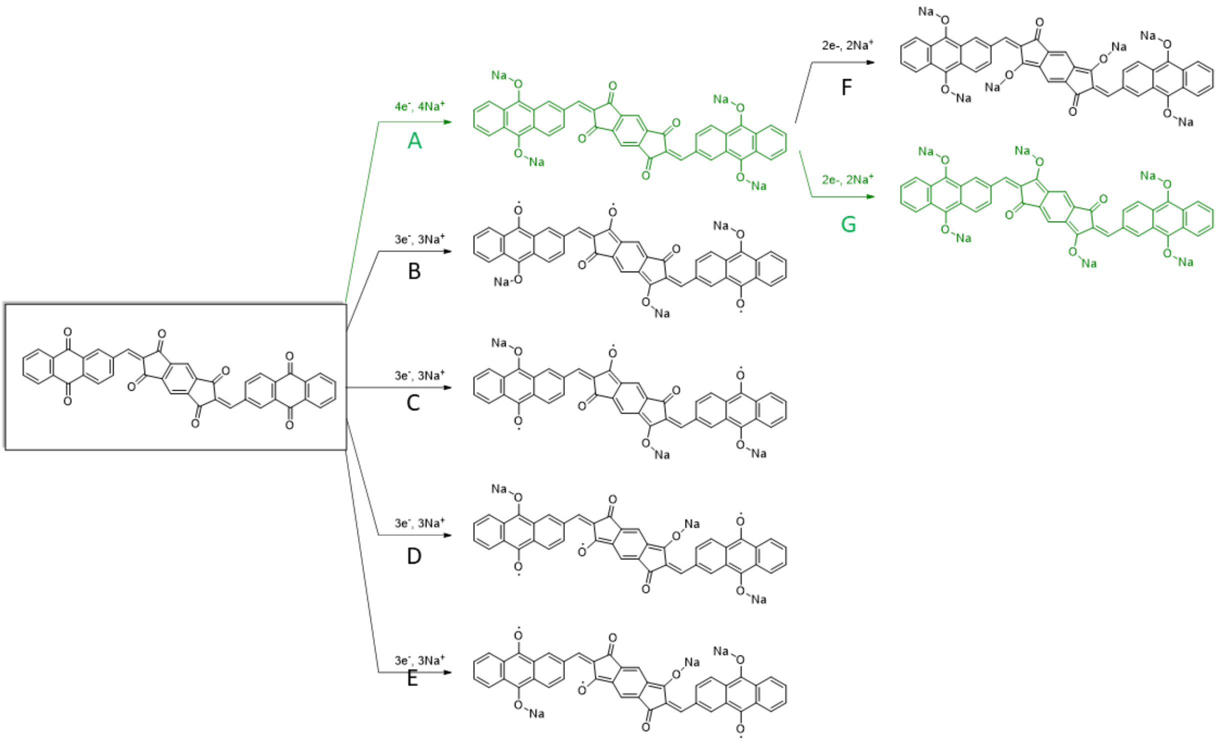

Pathway	Reduction	Resulting Energy (Hartree a.u.)	Optimised Structure
A	4e⁻, 4Na⁺	-2862.777554	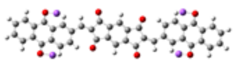
B	3e⁻, 3Na⁺	-2700.458314	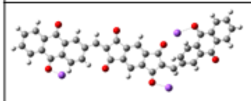
C	3e⁻, 3Na⁺	-2700.415146	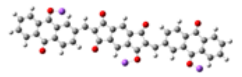
D	3e⁻, 3Na⁺	-2700.436772	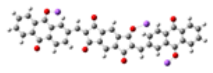
E	3e⁻, 3Na⁺	-2700.446509	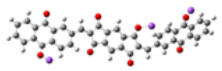
F	2e⁻, 2Na⁺	-3187.426067	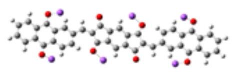
G	2e⁻, 2Na⁺	-3187.429611	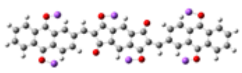

Figure S17: Comparison of reduction/sodium binding pathways of **BAQIT**. Sum of electronic and thermal free energies calculated via DFT calculations. The route highlighted in green displays lowest energy and theorized sodium binding mechanism.

5. References

1. Krief, P.; Becker, J. Y.; Ellern, A.; Khodorkovsky, V.; Neilands, O.; Shapiro, L. *s*-Indacene-1,3,5,7(2*H*,6*H*)-tetraone ('*Janus* dione') and 1,3-Dioxo-5,6-indane-dicarboxylic Acid: Old and New 1,3-Indandione Derivatives. *Synthesis*, 2004, **2004**, 2509-2512.
2. Akpinar, H.; Schlueter, J. A.; Lahti, P. M. 2-(9,10-Anthraquinon-2-yl)-4,4,5,5-tetramethyl-4,5-dihydro-1*H*-imidazole-3-oxide-1-oxyl: polymorphism in a conjugated anthraquinone-substituted nitronylnitroxide. *Chem. Commun.*, 2013, **49**, 3345-3347.
3. Xie, J.; Chen, W.; Long, G.; Gao, W.; Xu, Z. J.; Liu, M.; Zhang, Q., Boosting the performance of organic cathodes through structure tuning. *Journal of Materials Chemistry A* **2018,** *6* (27), 12985-12991.
4. Liang, Y.; Zhang, P.; Yang, S.; Tao, Z.; Chen, J., Fused Heteroaromatic Organic Compounds for High-Power Electrodes of Rechargeable Lithium Batteries. *Advanced Energy Materials* **2013,** *3* (5), 600-605.
5. Zhao, Q.; Wang, J.; Lu, Y.; Li, Y.; Liang, G.; Chen, J., Oxocarbon Salts for Fast Rechargeable Batteries. *Angewandte Chemie International Edition* **2016,** *55* (40), 12528-12532.
6. Yang, A.; Wang, X.; Lu, Y.; Miao, L.; Xie, W.; Chen, J., Core-shell structured 1,4-benzoquinone@TiO2 cathode for lithium batteries. *Journal of Energy Chemistry* **2018,** *27* (6), 1644-1650.
7. Teranishi, T.; Yoshikawa, Y.; Sakuma, R.; Hashimoto, H.; Hayashi, H.; Kishimoto, A.; Fujii, T., High-rate performance of ferroelectric BaTiO3-coated LiCoO2 for Li-ion batteries. *Applied Physics Letters* **2014,** *105* (14), 143904.
8. Gaussian 09, M. J. Frisch, G. W. Trucks, H. B. Schlegel, G. E. Scuseria, M. A. Robb, J. R. Cheeseman, G. Scalmani, V. Barone, G. A. Petersson, H. Nakatsuji, X. Li, M. Caricato, A. Marenich, J. Bloino, B. G. Janesko, R. Gomperts, B. Mennucci, H. P. Hratchian, J. V. Ortiz, A. F. Izmaylov, J. L. Sonnenberg, D. Williams-Young, F. Ding, F. Lipparini, F. Egidi, J. Goings, B. Peng, A. Petrone, T. Henderson, D. Ranasinghe, V. G. Zakrzewski, J. Gao, N. Rega, G. Zheng, W. Liang, M. Hada, M. Ehara, K. Toyota, R. Fukuda, J. Hasegawa, M. Ishida, T. Nakajima, Y. Honda, O. Kitao, H. Nakai, T. Vreven, K. Throssell, J. A. Montgomery, Jr., J. E. Peralta, F. Ogliaro, M. Bearpark, J. J. Heyd, E. Brothers, K. N. Kudin, V. N. Staroverov, T. Keith, R. Kobayashi, J. Normand, K. Raghavachari, A. Rendell, J. C. Burant, S. S. Iyengar, J. Tomasi, M. Cossi, J. M. Millam, M. Klene, C. Adamo, R. Cammi, J. W. Ochterski, R. L. Martin, K. Morokuma, O. Farkas, J. B. Foresman, and D. J. Fox, Gaussian, Inc., Wallingford CT, 2016.
